# *Mir221-* and *Mir222*-enriched adsc-exosomes mitigate PM exposure-exacerbated cardiac ischemia-reperfusion injury through the modulation of the BNIP3-MAP1LC3B-BBC3/PUMA pathway

**DOI:** 10.1080/15548627.2024.2395799

**Published:** 2024-09-08

**Authors:** Tzu-Lin Lee, Wen-Chi Shen, Ya-Chun Chen, Tsai-Chun Lai, Shu-Rung Lin, Shu-Wha Lin, I-Shing Yu, Yen-Hsiu Yeh, Tsai-Kun Li, I-Ta Lee, Chiang-Wen Lee, Yuh-Lien Chen

**Affiliations:** a Department of Anatomy and Cell Biology, College of Medicine, National Taiwan University, Taipei, Taiwan; b Department of Life Sciences, College of Life Sciences, National Chung Hsing University, Taichung, Taiwan; c The iEGG and Animal Biotechnology Center, National Chung Hsing University, Taichung, Taiwan; d Department of Bioscience Technology, College of Science, Chung Yuan Christian University, Taoyuan, Taiwan; e Center for Nanotechnology, Chung Yuan Christian University, Taoyuan, Taiwan; f Department of Clinical Laboratory Sciences and Medical Biotechnology, College of Medicine, National Taiwan University, Taipei, Taiwan; g Laboratory Animal Center, College of Medicine, National Taiwan University, Taipei, Taiwan; h Department and Graduate Institute of Microbiology, College of Medicine, National Taiwan University, Taipei, Taiwan; i Center for Biotechnology, National Taiwan University, Taipei, Taiwan; j Centers for Genomic and Precision Medicine, National Taiwan University, Taipei, Taiwan; k School of Dentistry, College of Oral Medicine, Taipei Medical University, Taipei, Taiwan; l Department of Orthopaedic Surgery, Chang Gung Memorial Hospital, Puzi, Chiayi, Taiwan; m Department of Nursing, Division of Basic Medical Sciences, and Chronic Diseases and Health Promotion Research Center Chang Gung University of Science and Technology, Puzi, Chiayi, Taiwan; n Research Center for Industry of Human Ecology and Research Center for Chinese Herbal Medicine, Chang Gung University of Science and Technology, Taoyuan, Taiwan

**Keywords:** ADSC-exosomes, cardiomyocyte, ischemia/reperfusion injury, *Mir221* and *Mir222*, mitophagy, particulate matter

## Abstract

Epidemiology has shown a strong relationship between fine particulate matter (PM) exposure and cardiovascular disease. However, it remains unknown whether PM aggravates myocardial ischemia-reperfusion (I/R) injury, and the related mechanisms are unclear. Our previous study has shown that adipose stem cell-derived exosomes (ADSC-Exos) contain high levels of *Mir221* and *Mir222*. The present study investigated the effects of PM exposure on I/R-induced cardiac injury through mitophagy and apoptosis, as well as the potential role of *Mir221* and *Mir222* in ADSC-Exos. Wild-type, *mir221-* and *mir222-*knockout (KO), and *Mir221-* and *Mir222-*overexpressing transgenic (TG) mice were intratracheally injected with PM (10 mg/kg). After 24 h, mice underwent left coronary artery ligation for 30 min, followed by 3 h of reperfusion (I/R). H9c2 cardiomyocytes were cultured under 1% O_2_ for 6 h, then reoxygenated for 12 h (hypoxia-reoxygenation [H/R]). PM aggravated I/R (or H/R) cardiac injury by increasing ROS levels and causing mitochondrial dysfunction, which increased the expression of mitochondrial fission-related proteins (DNM1L/Drp1 and MFF) and mitophagy-related proteins (BNIP3 and MAP1LC3B/LC3B) *in vivo* and *in vitro*. Treatment with ADSC-Exos or *Mir221-* and *Mir222-*mimics significantly reduced PM+I/R-induced cardiac injury. Importantly, ADSC-Exos contain *Mir221* and *Mir222*, which directly targets BNIP3, MAP1LC3B/LC3B, and BBC3/PUMA, decreasing their expression and ultimately reducing cardiomyocyte mitophagy and apoptosis. The present data showed that ADSC-Exos treatment regulated mitophagy and apoptosis through the *Mir221* and *Mir222*-BNIP3-MAP1LC3B-BBC3/PUMA pathway and significantly reduced the cardiac damage caused by PM+I/R. The present study revealed the novel therapeutic potential of ADSC-Exos in alleviating PM-induced exacerbation of myocardial I/R injury.

**Abbreviation:** ADSC-Exos: adipose-derived stem cell exosomes; AL: autolysosome; ATP: adenosine triphosphate; BBC3/PUMA: BCL2 binding component 3; BNIP3: BCL2/adenovirus E1B interacting protein 3; CASP3: caspase 3; CASP9: caspase 9; CDKN1B/p27: cyclin dependent kinase inhibitor 1B; CVD: cardiovascular disease; DCFH-DA: 2‘,7’-dichlorodihydrofluorescein diacetate; DHE: dihydroethidium; DNM1L/Drp1: dynamin 1-like; EF: ejection fraction; FS: fractional shortening; H/R: hypoxia-reoxygenation; I/R: ischemia-reperfusion; LDH: lactate dehydrogenase; MAP1LC3B/LC3B: microtubule-associated protein 1 light chain 3 beta; MFF: mitochondrial fission factor; miRNA: microRNA; NAC: N-acetylcysteine; OCR: oxygen consumption rate; PIK3C3/Vps34: phosphatidylinositol 3-kinase catalytic subunit type 3; PM: particulate matter; PRKAA1/AMPK: protein kinase AMP-activated catalytic subunit alpha 1; ROS: reactive oxygen species; SQSTM1/p62: sequestosome 1; TEM: transmission electron microscopy; TRP53/p53: transformation related protein 53; TUNEL: terminal deoxynucleotidyl transferase dUTP nick end labeling.

## Introduction

Increasing epidemiological evidence suggests that short- and long-term exposure to particulate matter (PM), especially fine particulate matter (PM2.5), can lead to the induction, progression, and worsening of cardiovascular disease (CVD) [1]. Inhalation of PM2.5 causes oxidative stress and inflammatory responses in the lungs, systemic circulation, and related organs/tissues (e.g., heart, vasculature, and adipocytes). Evidence from animal models suggests that atherosclerosis, hypertension, thrombosis, and heart failure associated with cardiovascular disease may be exacerbated by PM2.5 [2,3]. Coronary artery disease is the most common form of CVD. The medical strategy to treat coronary artery disease is timely reperfusion, such as percutaneous coronary intervention/PCI and coronary artery bypass grafting/CABG [4]. However, while reperfusion promotes blood flow recovery in the border zone of myocardial infarction and rescues myocardial cells, it also exacerbates cardiac ischemia-reperfusion (I/R) myocardial injury. The mortality of cardiac I/R injury has increased over the past few decades. Efforts have been made to elucidate the precise cellular and molecular mechanisms of cardiac I/R injury, such as the interaction between autophagy and apoptosis [5]. However, PM-related I/R-induced cardiac injury and its associated mechanisms, especially mitochondrial dysfunction, remain poorly understood.

Several mechanisms, including reactive oxygen species (ROS) overproduction, apoptosis, and imbalanced mitochondrial dynamics, have been proposed to contribute to cardiac I/R injury [6]. A previous study has reported that increased mitochondrial fission and decreased mitochondrial fusion are observed during cardiac I/R injury, resulting in cardiac damage [7]. Mitochondrial fission is regulated by DNM1L/Drp1 (dynamin 1-like) and MFF (mitochondrial fission factor). Furthermore, mitochondria are critical for maintaining normal cellular functions through mitophagy [8]. The role of mitophagy remains controversial. Mitophagy has beneficial and adverse effects on CVD [9]. Autophagy is thought to be cardioprotective during ischemia but detrimental during reperfusion [10,11]. BNIP3 (BCL2/adenovirus E1B interacting protein 3) mediates mitophagy through its cytoplasmic LIR granules, which interact with MAP1LC3B/LC3B (microtubule-associated protein 1 light chain 3 beta)-family proteins [12]. In addition, myocardial I/R injury induces MAPK/JNK activation, increases MFF activity, increases BNIP3 activity, promotes excessive mitochondrial fission, promotes mitophagy, and leads to cell death [13]. BNIP3 is activated upon ischemic stimulation and promotes cardiomyocyte death [14]. Furthermore, PM increases ROS levels and triggers cardiomyocyte apoptosis in AC16 cardiomyocytes [15]. Short-term exposure to PM triggers mitophagy, which leads to mitochondrial dysfunction, partially mediating vascular fibrosis [16]. There are currently no reports elucidating the correlation among myocardial damage, disruption of the fission-fusion process, and mitophagy caused by the exposure of PM combined with I/R or H/R.

Adipose-derived stem cells (ADSCs) secrete paracrine products, including cytokines, growth factors, RNA, and microRNAs, which can be delivered to target organs for repair [17]. As essential transporters of paracrine factors, exosomes play important roles in angiogenesis, immune regulation, and tissue regeneration [18]. Our previous studies have shown that exosomes from ADSCs protect skin flaps and the heart from ischemia/reperfusion injury [19,20]. Evidence suggests that miRNAs are involved in various biological processes, including myocardial mitochondrial ROS, inflammation, and apoptosis [21]. Some evidence suggests that *Mir221* and *Mir222* are key regulators of cardiovascular function and tissue metabolism [22]. In addition, *Mir221* and *Mir222* promotes migration and invasion, as well as inhibits autophagy and apoptosis [23]. *Mir222* has also been shown to be associated with particulate matter exposure alone [24]. In the present study, miRTarBase was used to predict the specific binding sites of *Mir221* and *Mir222* in the 3’ untranslated regions of *Bnip3* and *Lc3b*, suggesting that *Mir221* and *Mir222* may mediate mitophagy by regulating the *Bnip3* and *Lc3b* genes. Currently, there is limited knowledge regarding the involvement of *Mir221* and *Mir222* in the induction of cardiomyocyte apoptosis and mitophagy when PM and I/R (or H/R) are combined. The present study demonstrated that PM aggravates cardiac injury caused by I/R or H/R. In addition, *Mir221* and *Mir222* enrichment in ADSC-Exos affects ROS production, mitochondrial dynamics, and mitophagy, thereby reducing cardiac injury through the *Mir221* and *Mir222*-BNIP3-MAP1LC3B/LC3B-BBC3/PUMA pathway.

## Results

### Changes in ROS production and mitochondrial dynamics in vivo indicate that PM exposure aggravates I/R-induced cardiac injury

**Figure 1.**
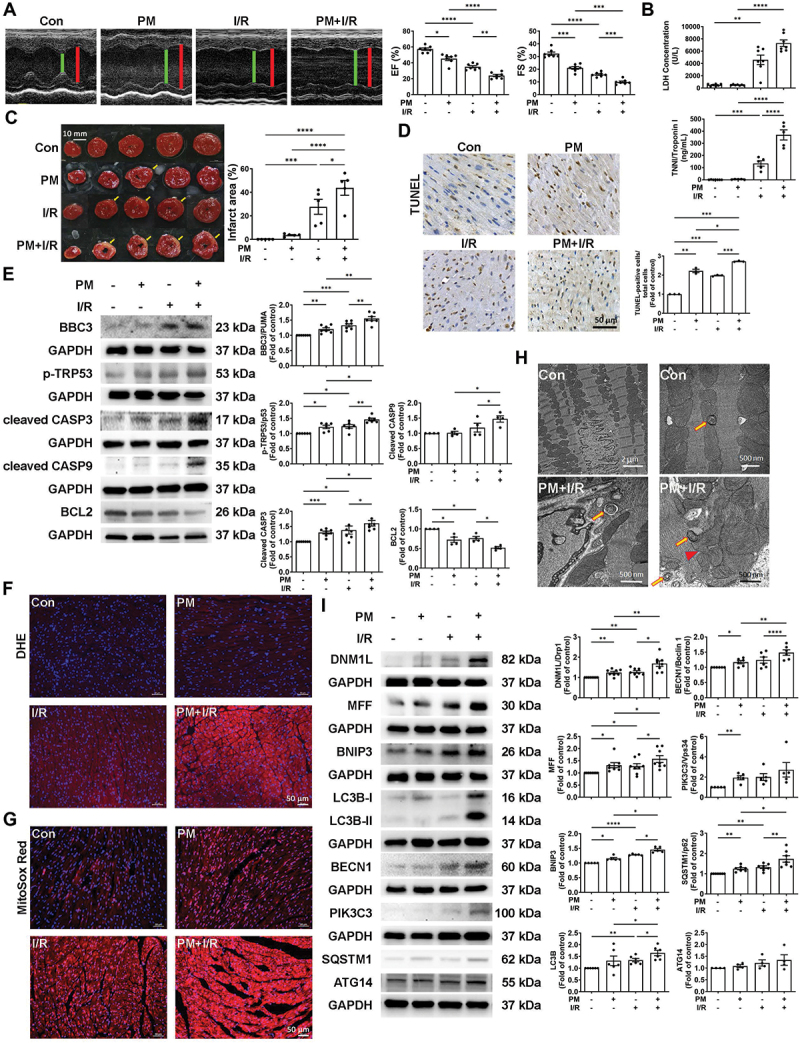
PM significantly impairs cardiac function and increases apoptosis in the hearts of C57BL/6J mice subjected to I/R. C57BL/6J mice were intratracheally injected with PM for 24 h, followed by 30 min of ischemia and 3 h of reperfusion. (A) images of the left ventricular end-systolic diameter (green line) and left ventricular end-diastolic diameter (red line) were obtained by echocardiography. EF and FS percentages were measured in control, PM, I/R, and PM+I/R mice (*n* = 7 mice per group). (B) cardiac injury was assessed by measuring plasma LDH and TNNI levels (*n* = 5-7 mice per group). (C) TTC staining was used to detect the ischemic area. The yellow arrows indicate the ischemic area (scale bar: 10 mm, *n* = 5 mice per group). (D) apoptotic cells were assessed by TUNEL assay (brown). Nuclei were counterstained by hematoxylin staining (blue). Scale bar: 50 μm; *n* = 3 mice per group. (E) Western blot analysis of the expression of apoptosis-related proteins (BBC3/PUMA, p-TRP53/p53, cl-CASP3, cl-CASP9, and BCL2) (*n* = 4-7). (F and G) intracellular and mitochondrial ROS were measured using DHE and MitoSOX red, respectively, and nuclei were stained blue with DAPI. Scale bar: 50 μm. (H) ultrastructural morphology observed via TEM. Autophagosome and mitochondrial fission are indicated by arrowheads and arrows, respectively. Scale bar: 2 μm or 500 nm. (I) Western blot analysis of the expression of mitochondrial fission-related proteins (DNM1L/Drp1 and MFF) and mitophagy-related proteins (BNIP3, MAP1LC3B/LC3B, BECN1/Beclin 1, PIK3C3/Vps34, SQSTM1/p62 and ATG14) (*n* = 5-8). The data are expressed as the mean ± SEM; one-way ANOVA.

Echocardiography was used to assess whether PM affects cardiac function in mice under I/R conditions. Representative echocardiographic images were obtained from control, PM-, I/R-, and PM+I/R-treated mice. Compared to control mice, mice treated with PM or I/R alone had significantly reduced ejection fraction (EF) and fractional shortening (FS) percentages, indicating impaired systolic contractility. Moreover, the EF and FS of the PM+I/R group were even lower than those of the PM and I/R groups (Figure 1A). In addition, the plasma levels of LDH (lactate dehydrogenase) and TNNI (troponin I), which are indicators of cardiac injury, were significantly elevated in the PM+I/R group (Figure 1B). Analysis of the ischemic area using TTC staining revealed that the white infarct area in the PM+I/R group was significantly greater than that in the PM and I/R groups (Figure 1C). The underlying mechanisms affecting the impairment of cardiac function caused by PM exposure in I/R-treated mice were investigated. No obvious histological damage was detected by hematoxylin and eosin staining (Figure S1). To investigate whether the combination of PM exposure and I/R directly leads to cardiomyocyte apoptosis, which causes cardiac dysfunction, TUNEL staining was used to detect the degree of cardiomyocyte apoptosis in mice treated with PM and I/R. The proportion of apoptotic cells was significantly greater in the PM+I/R group than in the PM or I/R alone group according to the TUNEL assay (Figure 1D). After PM+I/R treatment, the expression of BBC3/PUMA, p-TRP53/p53, cleaved CASP3 (caspase 3), and cleaved CASP9 (caspase 9) was significantly increased, whereas the expression of the BCL2 antiapoptotic protein was decreased (Figure 1E). I/R-induced cardiac injury is often caused by ROS production [6], and excessive ROS production results in mitochondrial dysfunction. In the present study, dihydroethidium (DHE) and MitoSOX Red were used to measure cellular and mitochondrial ROS, respectively. Cardiomyocytes subjected to I/R exhibited substantial ROS accumulation in cardiac tissue (Figures 1F, 1G, S2A, and S2B). Moreover, TEM analysis of heart tissue revealed mitochondrial ultrastructural changes in the hearts of PM+I/R-treated mice. In the control group, there were many normal mitochondria and myofilaments but few autophagosomes in the cytoplasm of cardiomyocytes. In contrast, the PM+I/R group exhibited many autophagosomes and mitochondrial fission (Figure 1H and S2C). To gain a more comprehensive understanding of mitochondrial dysfunction in PM+I/R hearts, the levels of mitochondrial fission-related proteins (DNM1L/Drp1 and MFF) and mitophagy-related proteins (BNIP3, MAP1LC3B/LC3B, BECN1/Beclin 1, PIK3C3/Vps34, SQSTM1/p62 and ATG14) were evaluated by western blot analysis. The expression levels of DNM1L/Drp1, MFF, BNIP3, MAP1LC3B/LC3B, BECN1/Beclin 1 and SQSTM1/p62 in the PM+I/R group were significantly higher than those in the PM alone and I/R alone treatment groups (Figure 1I). These results revealed that exposure to PM exacerbates I/R-induced cardiac diseases, increases ROS production, accelerates apoptosis, and promotes mitophagy.

Statistical significance was defined as *p < 0.05, **p < 0.01, and ***p < 0.001.

### PM exacerbates cardiomyocyte apoptosis through ROS generation under H/R conditions

**Figure 2.**
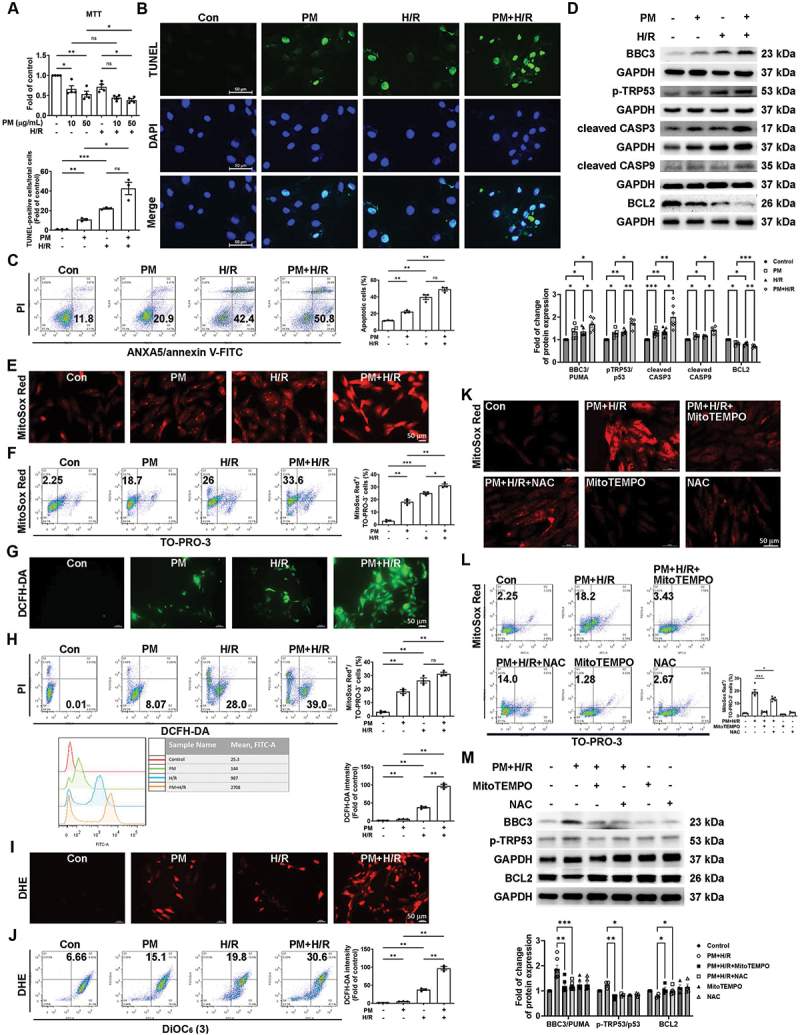
PM significantly exacerbates H/R-induced cardiomyocyte damage. H9c2 cells were pretreated with or without different concentrations of PM (10 or 50 μg/mL) for 6 h, followed by treatment with or without hypoxia for 6 h and reoxygenation for 12 h. (A) cell viability was assessed by MTT assay (*n* = 4). PM (50 μg/mL) was used in the following experiments. (B) a TUNEL assay was used to assess apoptosis. Nuclei were stained with DAPI (blue), and tunel-positive cells were indicated by green fluorescence (scale bar: 50 μm, *n* = 3). (C) flow cytometry was used to measure the number of apoptotic cells via ANXA5/annexin V-FITC-PI staining (*n* = 3). (D) the levels of apoptosis-related proteins were measured by western blot analysis (*n* = 4-7). (E) fluorescence microscopy was used to detect ROS production by MitoSOX red (1 μM) staining (scale bar: 50 μm, *n* = 4). (F) MitoSOX red (1 μM)/TO-PRO-3 (100 nM) staining was used to identify viable cells producing mitochondrial ROS (MitoSOX red-positive/TO-PRO-3-negative) by flow cytometry (*n* = 3). (G) by fluorescence microscopy, DCFH-DA (10 μM) was used to detect cytoplasmic H_2_O_2_ (scale bar: 50 μm, *n* = 3-5). (H) DCFH-DA/PI staining was used to examine viable cells producing cytoplasmic H_2_O_2_ (DCFH-DA-positive/PI-negative) by flow cytometry (*n* = 3). (I) DHE (5 μM) was used to detect cellular superoxide anions by fluorescence microscopy (scale bar: 50 μm, *n* = 4). (J) DHE/DiOC_6_(3) (90 nM) staining was used to examine viable cells producing cellular superoxide anions (DHE-positive/DiOC_6_(3)-positive) by flow cytometry (*n* = 3). (K-M) H9c2 cells were pretreated with MitoTEMPO (10 nM) or NAC (5 mM) for 1 h before exposure to PM+H/R. MitoSOX red was used to detect mitochondrial ROS production through fluorescence microscopy and flow cytometry. Western blot was used to detect the levels of cell apoptosis (scale bar: 50 μm, *n* = 4-7). The data are expressed as the mean ± SEM; one-way ANOVA.

To investigate the detailed mechanism by which PM exacerbates I/R-induced cardiac injury, a cell model was established by treating cells with different PM concentrations for 6 h, followed by 6 h of hypoxia and 12 h of reoxygenation (H/R). In the H/R model, cell viability decreased after 6 h of pretreatment of H9c2 cells with 10 or 50 µg/mL PM (Figure 2A). After treating H9c2 cells with 50 µg/mL PM and subjecting them to H/R (6 h/12 h), apoptosis was evaluated using TUNEL and ANXA5/annexin V-FITC-PI assays. TUNEL analysis revealed that apoptosis was significantly greater in the PM+H/R group than in the PM or H/R group (Figure 2B). The ANXA5/annexin V-FITC-PI assay showed that PM+H/R treatment accelerated apoptosis (Figure 2C). Compared to the PM or H/R group, the BBC3/PUMA, p-TRP53/p53, cleaved CASP3, and cleaved CASP9 expression was significantly increased in the PM+H/R group, whereas the BCL2 expression was significantly decreased in the PM+H/R group (Figure 2D). To study the effect of PM+H/R treatment, mitochondrial ROS was evaluated in H9c2 cells using MitoSOX Red and TO-PRO-3 double staining. Higher levels of mitochondrial ROS were detected in living cells (MitoSox Red-positive and TO-PRO-3-negative cells) under PM+H/R conditions (Figures 2E, 2F, and S2D). As shown by fluorescence microscopy and flow cytometry, PM+H/R treatment significantly increased the amount of cytoplasmic H_2_O_2_ in living cells (both PI-negative and DCFH-DA-positive cells) (Figures 2G, 2H, and S2E). Similarly, the DHE overlay resulting from fluorescence microscopy and flow cytometry showed that PM+H/R increased the production of cytoplasmic superoxide anions (DHE-positive and DiOC_6_(3)-positive cells) (Figures 2I, 2J, and S2F). The addition of MitoTEMPO (a mitochondrial antioxidant) and N-acetylcysteine (NAC, an antioxidant) inhibited the PM+H/R-induced increase in mitochondrial ROS observed by fluorescence microscopy and flow cytometry (Figures 2K, 2L, and S2G). Western blot showed that MitoTEMPO or NAC treatment reduced BBC3/PUMA and p-TRP53/p53 expression but increased BCL2 expression (Figure 2M). These results indicated that PM+H/R treatment increased ROS production *in vitro*, leading to apoptosis and cardiac damage.

Statistical significance was defined as *p < 0.05, **p < 0.01, and ***p < 0.001.

### PM exacerbates apoptosis in H/R-treated cardiomyocytes by worsening mitochondrial function and increasing mitophagy

**Figure 3.**
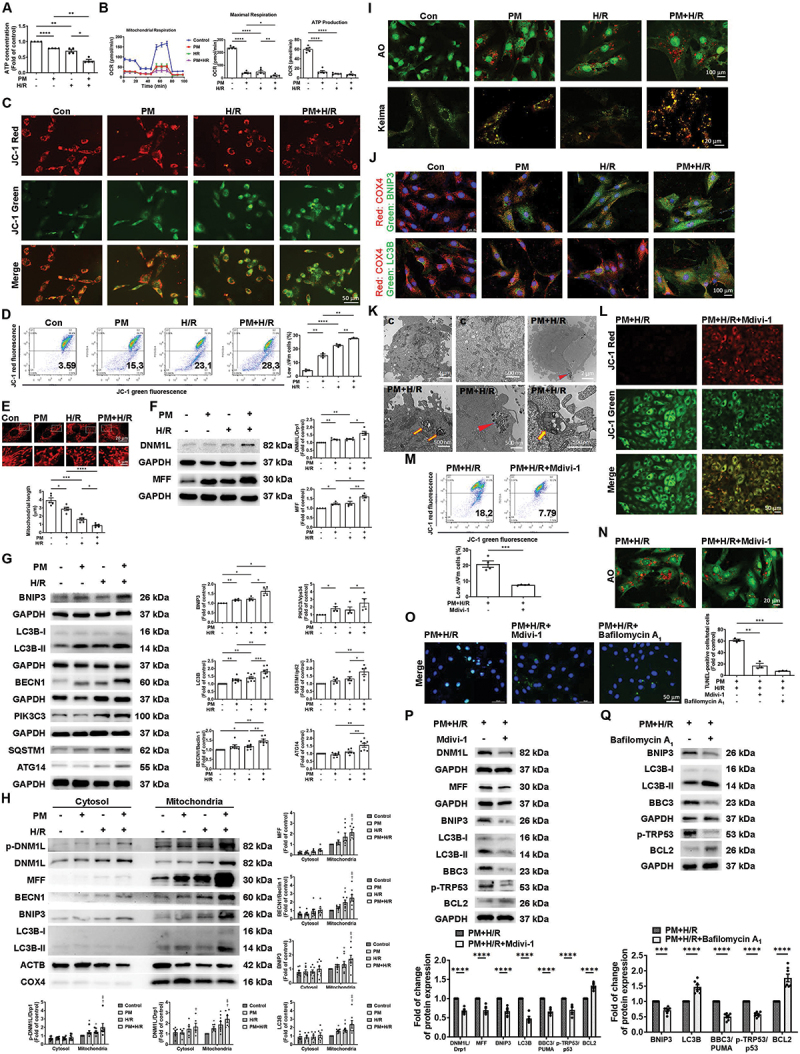
PM exacerbates mitochondrial dysfunction in H/R-treated cardiomyocytes. Cardiomyocytes were pretreated with or without 50 μg/mL PM and then exposed to hypoxia for 6 h, followed by reoxygenation for 12 h. (A) ATP levels were quantified (*n* = 4). (B) the OCR was determined using a seahorse metabolic analyzer. ATP levels and the maximal OCR were quantified (*n* = 5). (C and D) the δψm was determined by JC-1 staining under a fluorescence microscope and flow cytometry. High δψm and low δψm are shown in red and green, respectively (scale bar: 50 μm, *n* = 3). (E) mitochondrial length was measured with MitoTracker staining. The photo shown below is an enlargement of the rectangle above. Scale bar: 20 or 5 μm, as indicated in the panel, *n* = 5). (F) the levels of DNM1L/Drp1 and MFF were detected by western blot analysis (*n* = 4-5). (G) BNIP3, MAP1LC3B/LC3B, BECN1/Beclin 1, PIK3C3/Vps34, SQSTM1/p62 and ATG14 expression levels were examined by western blot analysis (*n* = 4-8). (H) the levels of p-DNM1L/DRP1, DNM1L/DRP1, MFF, BECN1/Beclin 1, BNIP3, and MAP1LC3B/LC3B in the cytoplasmic and mitochondrial fractions were examined by western blot analysis (*n* = 6-8). (I) ALs were observed via AO staining under a fluorescence microscope (scale bar: 100 μm, *n* = 4-5). Cardiomyocytes were transfected with the mitophagy reporter mitochondria-target Keima (mt-Keima) 48 h before treatment. Control cells showed green fluorescence, while pm+h/r-treated cells showed red fluorescence (scale bar: 20 μm, *n* = 6). (J) the colocalization of BNIP3 (green) or MAP1LC3B/LC3B (green) and COX4/COXIV (mitochondria, red) was observed by immunofluorescence microscopy (scale bar: 100 μm). (K) the ultrastructures of the PM and mitophagosomes were observed using TEM, as indicated in the panel. Scale bar: 2 μm or 500 nm). (L and M) the effect of mdivi-1 (10 µm) on the δψm was examined by the JC-1 assay (scale bar: 50 μm, *n* = 4). (N) the impact of mdivi-1 on ALs was examined by AO staining (scale bar: 20 μm). (O) effect of mdivi-1 and bafilomycin A_1_ (10 µm) on apoptosis according to the TUNEL assay (scale bar: 50 μm, *n* = 5). (P and Q) the impact of mdivi-1 and bafilomycin A_1_ treatment on the expression of mitochondrial fission-related, mitophagy-related, and apoptosis-related proteins was determined by western blot analysis (*n* = 5-7). The data are expressed as the mean ± SEM; one-way ANOVA.

Previous studies have demonstrated that impaired mitochondrial function or excessive mitochondrial fission can lead to cell death and cardiac injury [25,26]. The ATP concentration, oxygen consumption rate (OCR), and mitochondrial membrane potential (ΔΨm) are critical parameters of mitochondrial function. In the present study, the ATP concentration was significantly lower in the PM or H/R treatment group than in the control group, and the reduction was more significant in the PM+H/R combined treatment group than in the PM or H/R treatment group (Figure 3A). Mitochondrial function was assessed by measuring the OCR using a Seahorse metabolic analyzer. Compared with the control, PM or H/R significantly decreased the OCR (maximum) and ATP levels, which indicated that PM and H/R inhibited oxygen consumption and promoted extracellular acidification (Figure 3B). The effect of PM+H/R on the changes in the ΔΨm was determined using the JC-1 assay. PM+H/R caused a marked difference in the transition of fluorescence emission from red to green, which indicated a decrease in the ΔΨm (Figure 3C and S2H). Furthermore, flow cytometric analysis of JC-1 staining showed that PM+H/R treatment increased the number of cells with a low ΔΨm (JC-1 green positive and red negative) (Figure 3D). MitoTracker was used to examine the effect of PM+H/R on mitochondrial fission. The mitochondrial length in the PM+H/R-treated cells was significantly shorter than that in the other treated cells (Figure 3E). To further investigate the mechanism of PM+H/R regulation of mitochondrial fission, the levels of DNM1L/Drp1 and MFF were examined via western blot analysis. The expression of DNM1L/Drp1 and MFF was significantly greater in the PM and H/R groups than in the other treated groups, and PM+H/R treatment significantly enhanced the expression of DNM1L/Drp1 and MFF (Figure 3F). To investigate the mechanism underlying the PM+H/R effects on mitophagy, the expression of BNIP3, MAP1LC3B/LC3B, BECN1/Beclin 1, PIK3C3/Vps34, SQSTM1/p62 and ATG14 was evaluated by western blot analysis. PM+H/R increased the expression of BNIP3, MAP1LC3B/LC3B, BECN1/Beclin 1, SQSTM1/p62, and ATG14 proteins compared to the PM or H/R-alone-treated groups (Figure 3G). The present study investigated whether PM+H/R affects the translocation of *p*- DNM1L/Drp1, DNM1L/Drp1, MFF, BECN1/Beclin 1, BNIP3, and MAP1LC3B/LC3B from the cytoplasm to mitochondria in cardiomyocytes. Compared to PM- or H/R-treated cells, treatment with PM+H/R significantly increased the expression of these proteins in mitochondria (Figure 3H). The effect of PM on mitophagy in H9c2 cells under H/R conditions was verified by acridine orange (AO) staining, which indicated that there was an increase in the number of autolysosomes (AL) in PM+H/R-treated cells (Figure 3I and S2I). PM+H/R-treated cells also showed an increase in mitophagy, as shown by mitochondria-targeted mt-Keima-Red assay (Figure 3I). Fluorescence staining showed that PM treatment increased the localization of BNIP3 and MAP1LC3B/LC3B in the mitochondria of H/R-treated cells (Figure 3J). Normal mitochondria were observed in the cytoplasm of cardiomyocytes in the control group, while several autophagosomes and PM particles were observed in the cytoplasm of cardiomyocytes in the PM+H/R group (Figure 3K and S2J). Moreover, Mdivi-1, a mitochondrial fission inhibitor, reduced PM+H/R-induced mitochondrial dysfunction, as shown by the JC-1 assay (Figures 3L, 3M, and S2K). PM+H/R combined treatment increased ALs, while Mdivi-1 treatment inhibited red fluorescence by AO staining (Figure 3N). The TUNEL assay demonstrated that treatment with Mdivi-1 and bafilomycin A_1_ significantly reduced PM+H/R-induced apoptosis (Figure 3O). PM+H/R exposure increased the expression of fission-related proteins (DNM1L/Drp1 and MFF), mitophagy-related proteins (MAP1LC3B/LC3B and BNIP3), and apoptosis-related proteins (BBC3/PUMA and p-TRP53/p53) but decreased the expression of BCL2. However, Mdivi-1 treatment reversed these changes in protein expression (Figure 3P). Similarly, bafilomycin A_1_ treatment reversed the effect of PM+H/R on the expression of mitophagy- and apoptosis-related proteins (Figure 3Q). These findings indicated that PM+H/R treatment affects mitochondrial function, mitophagy, and mitochondrial fission, suggesting a potential role for these mechanisms in the cellular response to PM+H/R-induced cardiac injury.

Statistical significance was defined as *p < 0.05, **p < 0.01, and ***p < 0.001.

### PM+H/R significantly reduces Mir221 and Mir222 expression, while Mir221- and Mir222-enriched ADSC-Exos reduce pm+h/r-induced mitophagy and apoptosis

**Figure 4.**
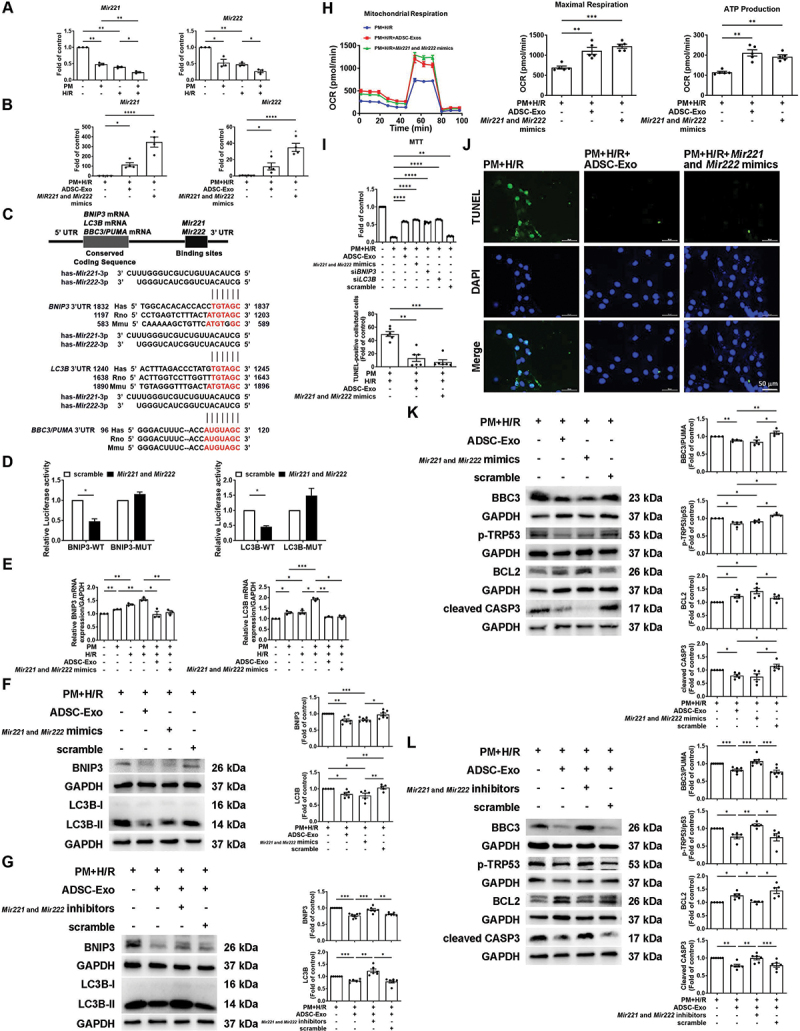
*Mir221* and *Mir222* regulate the expression of *Bnip3* and *Lc3b*, causing PM to aggravate myocardial H/R injury. H9c2 cells were transfected with *Mir221-* and *Mir222-*mimics or inhibitors for 24 h, pretreated with PM for 6 h, and subjected to H/R. (A) *Mir221* and *Mir222* expression in various treatment groups was assessed using qPCR (*n* = 3). (B) the impact of ADSC-Exos or *Mir221-* and *Mir222-*mimics on *Mir221* and *Mir222* expression in pm+h/r-treated cells was assessed by qPCR (*n* = 4). (C) schematic diagram depicting the binding of *Mir221* and *Mir222* to the 3’ UTRs of *Bnip3*, *Lc3b*, and *Bbc3/Puma* target genes. (D) luciferase activities of the *Bnip3* and *Lc3b* reporters in the *Mir221-* and *Mir222-*mimics and scramble groups were measured (*n* = 4). (E) effects of ADSC-Exos or *Mir221-* and *Mir222-*mimics on *Bnip3* and *Lc3b* mRNA expression in pm+h/r-treated cardiomyocytes (*n* = 3). (F and G) the effects of ADSC-Exos, *Mir221-* and *Mir222-*mimics (F), and *Mir221-* and *Mir222-*inhibitors (G) on the expression of mitophagy-related proteins were detected by western blot analysis (*n* = 5-7). (H) effects of ADSC-Exos or *Mir221-* and *Mir222-*mimics on the OCR in cardiomyocytes treated with PM+H/R (*n* = 5). (I) effect of ADSC-Exos, *Mir221-* and *Mir222-*mimics, si*BNIP3*, and si*LC3B* on the viability of pm+h/r-treated H9c2 cells (*n* = 5). (J) the impact of ADSC-Exos and *Mir221-* and *Mir222-*mimics on apoptosis in pm+h/r-treated cardiomyocytes was assessed via a TUNEL assay (scale bar: 50 μm, *n* = 6). (K and L) the effects of ADSC-Exos, *Mir221-* and *Mir222-*mimics (K), and *Mir221-* and *Mir222-*inhibitors (L) on the expression of apoptosis-related proteins were assessed by western blot analysis (*n* = 4-7). The data are expressed as the mean ± SEM; one-way ANOVA.

**Figure 5.**
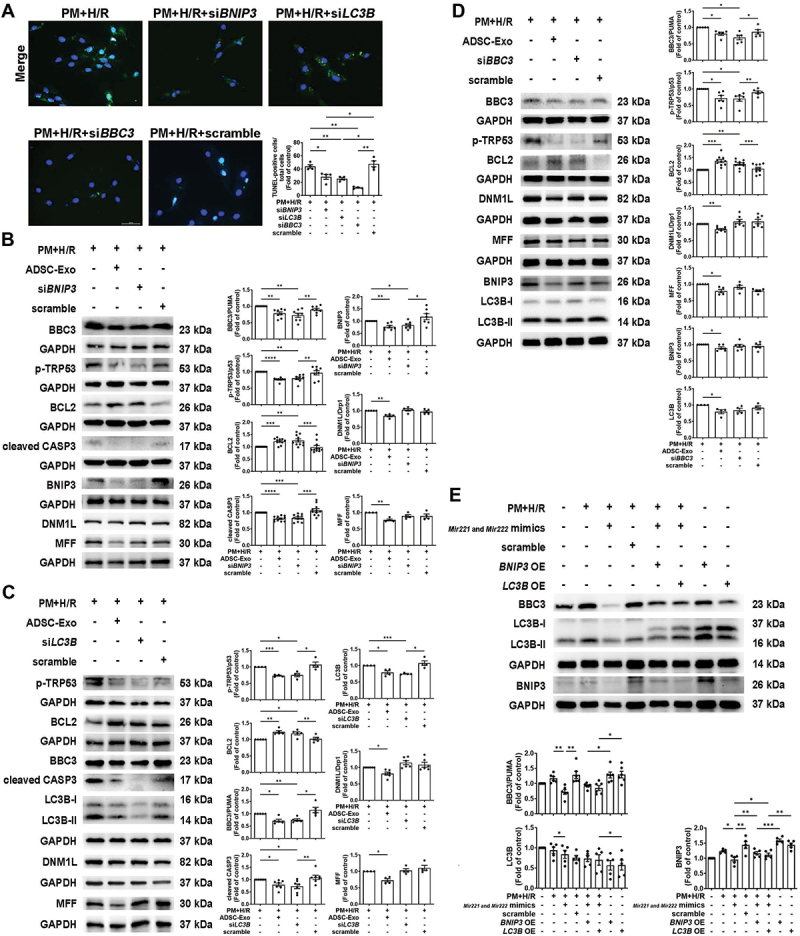
Effects of *Bnip3*, *Lc3b*, and *Bbc3/Puma* downregulation on pm+h/r-induced mitochondrial fission, mitochondrial autophagy, and apoptosis. (A) the effects of the downregulation of *Bnip3*, *Lc3b*, and *Bbc3/Puma* on cell apoptosis in pm+h/r-treated cardiomyocytes were examined by a TUNEL assay (scale bar: 50 μm, *n* = 4). (B and C) the effects of the downregulation of *Bnip3* (B) and *Lc3b* (C) on the expression of mitochondrial fission-related, mitophagy-related, and apoptosis-related proteins in cells exposed to PM+H/R, was determined by western blot analysis (*n* = 4-11). (D) western blot analysis of the effects of *Bbc3/Puma* downregulation on the expression of mitochondrial fission- and mitophagy-related proteins in cells exposed to PM+H/R (*n* = 5-10). (E) H9c2 cells were transfected with *Mir221* and *Mir222* or with *Bnip3* and *Lc3b* overexpression, and then exposed to PM+H/R conditions. The expression levels of BBC3/PUMA, MAP1LC3B/LC3B, and BNIP3 proteins were assessed by western blot. The data are expressed as the mean ± SEM; one-way ANOVA.

We have previously demonstrated that an abundance of *Mir221* and *Mir222* in ADSC-Exos reduces injury caused by I/R [20]. To explore whether *Mir221* and *Mir222* regulates PM+H/R-induced mitophagy and apoptosis, the levels of *Mir221* and *Mir222* were assessed by qPCR. Compared to the control group, the expression of *Mir221* and *Mir222* was significantly downregulated in the PM and H/R treatment groups, while treatment with PM+H/R further decreased the levels of *Mir221* and *Mir222* (Figure 4A). However, the addition of ADSC-Exos increased the levels of *Mir221* and *Mir222* in PM+H/R-treated H9c2 cells (Figure 4B). Compared to H9c2 cells and ADSCs, ADSC-Exos contained higher levels of *Mir221* and *Mir222* (Figure S3A). To elucidate the effects of *Mir221* and *Mir222* on mitophagy- and apoptosis-related proteins, target prediction was performed with miRTarBase, which predicted the *Mir221-* and *Mir222-*binding site in the 3’-UTRs of *Bnip3*, *Lc3b*, and *Bbc3/Puma* (Figure 4C). We have previously demonstrated that *Mir221* and *Mir222* target *Bbc3/Puma* and regulate apoptosis [27]. To determine whether *Mir221* and *Mir222* target the 3’ UTRs of *Bnip3* and *Lc3b*, the luciferase activity of reporter genes (*Bnip3* and *Lc3b*) was reduced in cells transfected with *Mir221-* and *Mir222-*mimics. Additionally, no significant difference was observed in cells transfected with *Bnip3*- and *Lc3b*-MUT, suggesting that *Mir221* and *Mir222* directly target *Bnip3* and *Lc3b* (Figure 4D). PM+H/R stimulation increased the mRNA and protein levels of *Bnip3* and *Lc3b*, but treatment with ADSC-Exos or *Mir221-* and *Mir222-*mimics decreased the mRNA and protein expression levels of *Bnip3* and *Lc3b* (Figures 4E,F). Compared to the PM+H/R+ADSC-Exo group, the expression of BNIP3 and LC3B was increased in the PM+H/R+ADSC-Exos+*Mir221-* and *Mir222-*inhibitors group (Figure 4G). A Seahorse analyzer was used to examine the efficiency of mitochondrial respiration and mitochondrial function. Treatment with ADSC-Exos and *Mir221-* and *Mir222-*mimics increased the OCR, ATP production, and maximal respiration in PM+H/R-treated cells, which indicated that ADSC-Exos and overexpression of *Mir221* and *Mir222* protect against PM+H/R-induced mitochondrial dysfunction (Figure 4H). Compared to treatment with PM+H/R, MTT assays showed an increase in cell viability after treatment with ADSC-Exos and *Mir221-* and *Mir222-*mimics (Figure 4I). The present study then investigated whether restoring *Mir221* and *Mir222* expression in the presence of PM+H/R alleviates apoptosis. TUNEL assays revealed that ADSC-Exos administration and *Mir221-* and *Mir222-*mimics transfection significantly reduced PM+H/R-induced apoptosis (Figure 4J). In addition, treatment with ADSC-Exos and *Mir221-* and *Mir222-*mimics reduced the effects of PM+H/R-induced upregulation of apoptosis-related proteins (BBC3/PUMA, p-TRP53/p53, and cleaved CASP3) and downregulation of the anti-apoptosis protein BCL2. Conversely, treatment with *Mir221-* and *Mir222-*inhibitors diminished the therapeutic function of ADSC-Exos in reducing apoptosis (Figures 4K,L). To further test the roles of *Bnip3* and *Lc3b* in regulating apoptosis and cell viability, siRNAs targeting *Bnip3* and *Lc3b* were used to knockdown their expression. TUNEL and MTT assays revealed that si*BNIP3* and si*LC3B* reduced cell apoptosis and increased cell viability compared to the PM+H/R group (Figures 5A and 4I). In PM+H/R-treated H9c2 cells, si*BNIP3* and si*LC3B* reduced the expression of BNIP3 and MAP1LC3B/LC3B, respectively, thereby reducing the expression of BBC3/PUMA, p-TRP53/p53 and cleaved CASP3, but increasing the expression of BCL2 (Figures 5B,C). Notably, si*BNIP3* and si*LC3B* effectively reduced the levels of apoptosis-related proteins but had no significant effect on fission-related proteins (DNM1L/Drp1 and MFF) under PM+H/R condition. Moreover, *Bbc3/Puma* knockdown did not affect the levels of BNIP3 and MAP1LC3B/LC3B under PM+H/R condition (Figure 5D). Furthermore, to validate the comprehensive mechanism involving *Mir221* and *Mir222*, we simultaneously overexpressed *Lc3b* and *Bnip3* and transfected *Mir221* and *Mir222*. We then observed the protein levels of BBC3/PUMA, MAP1LC3B/LC3B, and BNIP3 under PM+H/R conditions. western blot analysis demonstrated that *Mir221-* and *Mir222-*transfection reduced PM+H/R-induced BBC3/PUMA levels compared to scramble transfection (Figure 5E). However, simultaneous transfection of *Mir221* and *Mir222* and overexpression of *Bnip3* or *Lc3b* showed no reduction in PM+H/R-induced BBC3/PUMA levels compared to the scramble group.

Statistical significance was defined as *p < 0.05, **p < 0.01, and ***p < 0.001.

Statistical significance was defined as *p < 0.05, **p < 0.01, and ***p < 0.001. [Change labels to “si*BBC3*” in panels A and D.]

To investigate whether other signal transduction molecules are involved in PM+I/R-enhanced apoptosis, an AKT pathway phosphorylation array was used to identify specific pathways. PM+H/R treatment induced the upregulation of apoptotic proteins, such as PRKAA1/AMPK, CDKN1B/p27, and p-TRP53/p53 (Figure S4A). Western blot analysis identified significant changes in p-PRKAA1/AMPK and p-CDKN1B/p27 between PM+H/R-treated cells and ADSC-Exos-treated cells (Figure S4B). To elucidate whether ADSC-Exos affect apoptosis in PM+H/R-treated H9c2 cells via the PRKAA1/AMPK-CDKN1B/p27 pathway, H9c2 cells were treated with a CDKN1B/p27 inhibitor (SKPin C1) and an PRKAA1/AMPK inhibitor (compound C). SKPin C1 and compound C reduced the expression of BBC3/PUMA and p-TRP53/p53 but increased the expression of BCL2 in PM+H/R-treated H9c2 cells (Figure S4C). TUNEL assays indicated that SKpin C1 and compound C treatment significantly reduced apoptosis (Figure S4D). These results showed that treatment with ADSC-Exos reduces PM+H/R-induced mitophagy and apoptosis through the BNIP3-MAP1LC3B/LC3B-BBC3/PUMA pathway, and this process is regulated by *Mir221* and *Mir222*. In addition, the PRKAA1/AMPK-CDKN1B/p27 pathway is also involved in PM+H/R-induced apoptosis.

### ADSC-Exos attenuate pm+h/r-induced apoptosis by reducing ROS production, mitochondrial fission, and mitophagy

**Figure 6.**
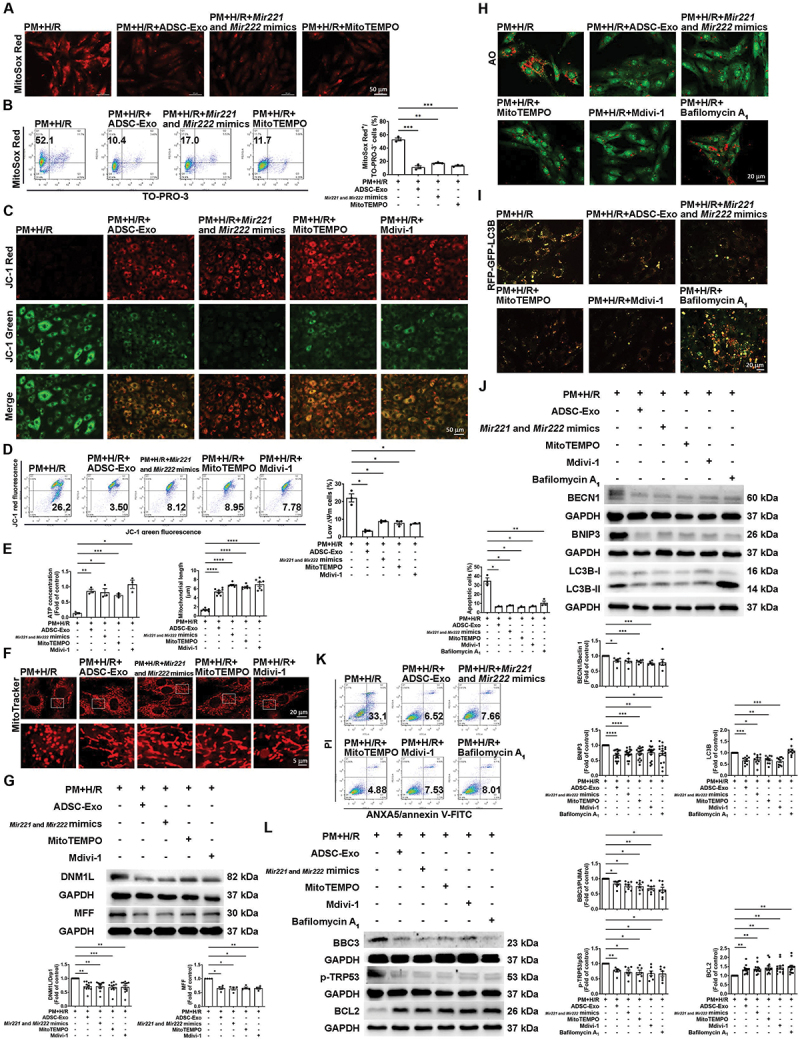
*Mir221* and *Mir222* in ADSC-Exos reduce mitochondrial ROS levels, mitochondrial fission, mitophagy, and apoptosis in cardiomyocytes exposed to PM+H/R. H9c2 cells were treated with ADSC-Exos (2 µg/mL) or transfected with *Mir221-* and *Mir222-*mimics for 24 h, followed by treatment with PM (50 µg/mL) and H/R (6 h/12 h). (A and B) the effects of ADSC-Exos, *Mir221-* and *Mir222-*mimics, and MitoTEMPO treatment on mitochondrial ROS were evaluated via MitoSOX red staining via fluorescence microscopy and flow cytometry (scale bar: 50 μm, *n* = 3). (C and D) the effects of ADSC-Exos, *Mir221-* and *Mir222-*mimics, MitoTEMPO, and mdivi-1 treatment on δψm were assessed by JC-1 staining under fluorescence microscopy and flow cytometry (scale bar: 50 μm, *n* = 3). (E) ATP production was assessed using an ATP assay (*n* = 3). (F) mitochondrial length was assessed by a MitoTracker assay (scale bar: 20 or 5 μm, *n* = 7). (G) the expression of DNM1L/Drp1 and MFF was assessed by western blot analysis (*n* = 4-11). (H) the effects of treatment with ADSC-Exos, *Mir221-* and *Mir222-*mimics, MitoTEMPO, mdivi-1, and bafilomycin A_1_ on AL accumulation was determined by AO staining (scale bar: 20 μm). (I) cardiomyocytes were transduced with RFP-GFP-LC3 lentivirus for 72 h. The lentivirus allowed the distinction of autophagosomes (GFP+ and RFP+; yellow plots) and ALs (GFP− and RFP+; red plots), as GFP fluorescence was quenched in the acidic ALs. The effects of ADSC-Exos, *Mir221-* and *Mir222-*mimics, MitoTEMPO, mdivi-1, and bafilomycin A_1_ on the formation of autophagosomes and ALs assessed by RFP-GFP-LC3 staining (scale bar: 20 μm, *n* = 10). (J) the effects of treatment with ADSC-Exos, *Mir221-* and *Mir222-*mimics, MitoTEMPO, mdivi-1, and bafilomycin A_1_ on the expression of mitophagy-related proteins were examined by western blot analysis (*n* = 6-17). (K and L) the effects of ADSC-Exos, *Mir221-* and *Mir222-*mimics, MitoTEMPO, mdivi-1, and bafilomycin A_1_ on cell apoptosis were assessed by ANXA5/annexin V-FITC-PI flow cytometry and western blot analysis (*n* = 3-13). The data are expressed as the mean ± SEM; one-way ANOVA.

Mitochondrial ROS production was measured under ADSC-Exos treatment, *Mir221-* and *Mir222-*mimics transfection, and MitoTEMPO treatment conditions using MitoSOX Red staining. Fluorescence microscopy and flow cytometry indicated that these treatments reduced the MitoSox Red fluorescence intensity in PM+H/R-treated cardiomyocytes (Figures 6A, 6B, and S2L), which indicated that ADSC-Exos and *Mir221-* and *Mir222-*mimics reduce the mitochondrial ROS levels in PM+H/R-treated H9c2 cells. JC-1 staining assessed by fluorescence microscopy and flow cytometry showed that PM+H/R treatment increased the number of cells with a low ΔΨm. Conversely, treatment with ADSC-Exos, *Mir221-* and *Mir222-*mimics, MitoTEMPO, or Mdivi-1 reversed the decrease in the ΔΨm induced by PM+H/R (Figures 6C, 6D, and S2M). Treatment of H9c2 cells with ADSC-Exos, *Mir221-* and *Mir222-*mimics, MitoTEMPO, and Mdivi-1 significantly increased ATP production in PM+H/R-treated cardiomyocytes (Figure 6E). Moreover, the mitochondrial length was shortened in PM+H/R-treated cardiomyocytes, and it was increased by ADSC-Exos, *Mir221-* and *Mir222-*mimics, MitoTEMPO, and Mdivi-1 (Figure 6F). Western blot analysis revealed that the above treatments also significantly reduced the levels of DNM1L/Drp1 and MFF (Figure 6G). To further confirm the effects of ADSC-Exos and *Mir221-* and *Mir222-*mimics on mitophagy after PM+H/R injury, AO staining, RFP-GFP-LC3B staining, and mitophagy-related proteins expression levels were measured. PM+H/R treatment increased the number of autophagosomes, whereas treatment with ADSC-Exos, *Mir221-* and *Mir222-*mimics, MitoTEMPO, or Mdivi-1 reduced the number of autophagosomes (Figure 6H and S2N). RFP-GFP-LC3B was used to visualize autophagosomes and ALs (yellow dots and red dots, respectively). Treatment with ADSC-Exos and *Mir221-* and *Mir222-*mimics reduced mitophagy (Figure 6I and S2O), as indicated by the reduced number of autophagosomes visualized by RFP-GFP-LC3. In addition, bafilomycin A_1_, which functions as a blocker of AL formation, showed an accumulation of autophagosomes under PM+H/R condition. Western blot results showed the addition of bafilomycin A_1_ reduced PM+H/R-increased BECN1/Beclin 1, BNIP3 and MAP1LC3B/LC3B levels (Figure 6J). We also treated the cells with MitoTEMPO, Mdivi-1, and bafilomycin A_1_ and examine their role in PM+H/R-induced apoptosis. ANXA5/annexin V-FITC-PI assay demonstrated that these treatments significantly decreased the number of apoptotic cells (Figure 6K), and these treatments also reduced the expression of the BBC3/PUMA and p-TRP53/p53 apoptotic proteins but increased the expression of BCL2 (Figure 6L). These results indicated that ROS trigger mitochondrial fission, mitophagy, and apoptosis in PM+H/R-treated cardiomyocytes, while ADSC-Exos inhibit ROS, mitochondrial fission, and mitophagy to attenuate cell apoptosis.

Statistical significance was defined as *p < 0.05, **p < 0.01, and ***p < 0.001.

### Mir221 and Mir222 attenuate pm+i/r-induced cardiac injury in WT and mir221- and mir222-ko mice and has a similar effect on TG mice

**Figure 7.**
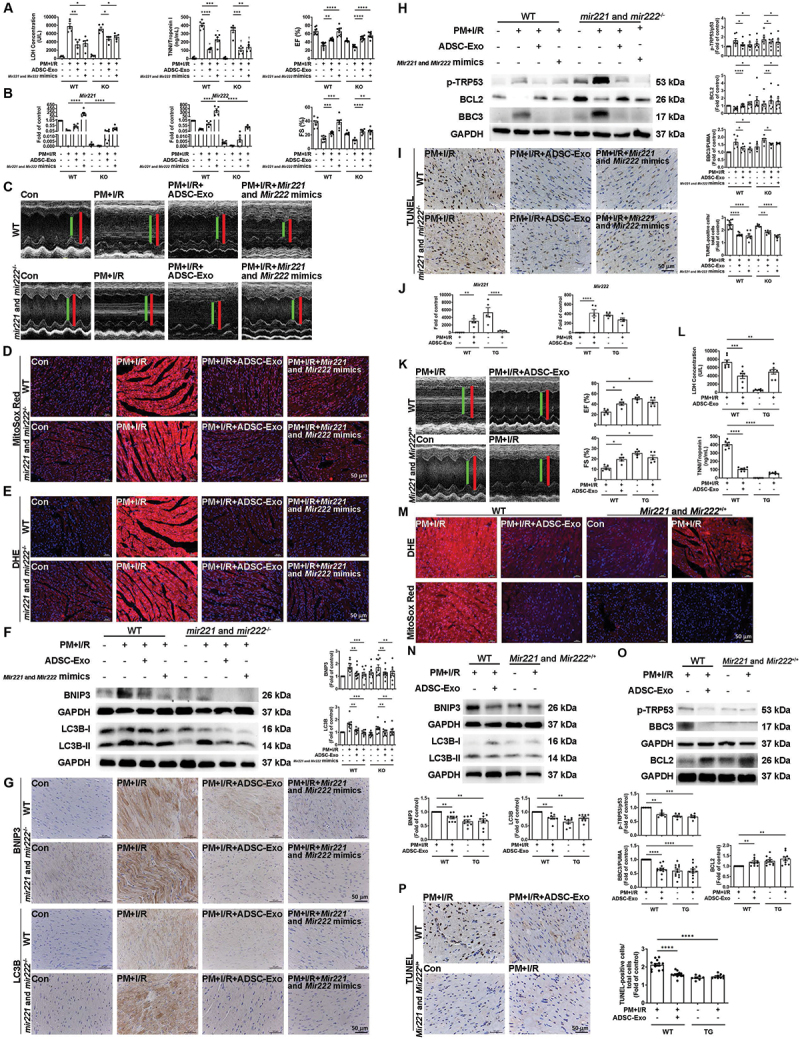
*Mir221* and *Mir222* decrease pm+i/r-induced cardiac damage in WT, *mir221-* and *mir222-*ko, and *Mir221-* and *Mir222-*tg mice. WT or *mir221-* and *mir222-*ko mice were preconditioned with PM for 24 h. Then, 25 min after occlusion, ADSC-Exos (100 μg of protein in 50 μL) or *Mir221-* and *Mir222-*mimics (100 nM) were uniformly intramuscularly injected into the left ventricular marginal zone. The effects of ADSC-Exos and *Mir221-* and *Mir222-*mimics on (A) plasma LDH and TNNI levels (*n* = 5 mice per group), as well as (B) *Mir221* and *Mir222* expression (*n* = 4-7 mice per group), were measured by qPCR. (C) cardiac function was measured via echocardiography (*n* = 7-9 mice per group). (D and E) ROS levels were measured via MitoSOX red staining and DHE staining (scale bar: 50 μm, *n* = 3-7). (F) BNIP3 and MAP1LC3B/LC3B expression was measured via western blot analysis (*n* = 9-10 mice per group). (G) BNIP3 and MAP1LC3B/LC3B expression was measured via immunohistochemistry (scale bar: 50 μm, *n* = 3-8). (H) the expression of apoptosis-related proteins was evaluated by western blot analysis. (I) the number of apoptotic cells was measured by a TUNEL assay (scale bar: 50 μm, *n* = 5-11 mice per group). To investigate the impact of *Mir221* and *Mir222* expression on pm+i/r-induced cardiac damage, *Mir221-* and *Mir222-*overexpression TG mice were preconditioned with PM for 24 h, followed by myocardial I/R. The results were compared to those of PM+I/R WT mice and PM+I/R+ADSC-Exos WT mice. (J) qPCR was performed to measure *Mir221* and *Mir222* expression (*n* = 5 mice per group). (K) echocardiography was used to assess heart function (*n* = 5 mice per group). (L) LDH and TNNI levels in plasma were measured (*n* = 6-7 mice per group). (M) ROS expression was detected using MitoSOX red and DHE staining (scale bar: 50 μm, *n* = 3-7). (N) western blot analysis was used to evaluate the mitochondrial autophagy-related protein levels (*n* = 8-9 mice per group). (O) western blot analysis was used to evaluate the apoptosis-related protein levels (*n* = 6-12 mice per group). (P) a TUNEL assay was used to quantify apoptotic cell numbers (scale bar: 50 μm, *n* = 7-13 mice per group). The data are expressed as the mean ± SEM; one-way ANOVA.

To elucidate whether ADSC-Exos protect mouse hearts from PM+I/R-induced damage through a *Mir221-* and *Mir222-*dependent mechanism, C57BL/6 (WT) and *mir221-* and *mir222-*KO mice were preconditioned with PM for 24 h. After 25 min of occlusion, ADSC-Exos and *Mir221-* and *Mir222-*mimics were intramuscularly injected into the anterior wall, followed by 3 h of reperfusion. LDH and TNNI were significantly increased in the I/R and PM+I/R groups. Treatment with ADSC-Exos or *Mir221-* and *Mir222-*mimics significantly reduced the PM+I/R-induced release of LDH and TNNI (Figure 7A). Treatment of WT and *mir221-* and *mir222-*KO mice with ADSC-Exos or *Mir221-* and *Mir222-*mimics increased *Mir221* and *Mir222* expression in the PM+I/R condition (Figure 7B). Treatment with ADSC-Exos or *Mir221-* and *Mir222-*mimics increased the EF and FS percentages in WT and *mir221-* and *mir222-*KO mice (Figure 7C). In contrast, ADSC-Exos collected from ADSCs transfected with *Mir221-* and *Mir222-*inhibitors failed to improve PM+I/R-induced cardiac dysfunction (Figure S5). The effect of ADSC-Exos on ROS production in cardiac tissue was further assessed. Treatment with ADSC-Exos or *Mir221-* and *Mir222-*mimics significantly reduced cytoplasmic and mitochondrial ROS generation, as detected by DHE and MitoSox Red staining (Figures 7D, 7E, S2P, and S2Q). In addition, PM+I/R increased the expression of BNIP3 and MAP1LC3B/LC3B, while ADSC-Exos and the *Mir221-* and *Mir222-*mimics reduced BNIP3 and MAP1LC3B/LC3B expression, as determined by western blot analysis and immunohistochemistry (Figures 7F, 7G, S2R and S2S). After PM+I/R treatment, BBC3/PUMA and p-TRP53/p53 expression significantly increased, while BCL2 expression decreased in WT as well as *mir221-* and *mir222-*KO mice, while ADSC-Exos or *Mir221-* and *Mir222-*mimics reversed these changes (Figure 7H). Furthermore, the TUNEL assay demonstrated that treatment with ADSC-Exos or *Mir221-* and *Mir222-*mimics reduced cardiac apoptosis (Figure 7I).

**Figure 8.**
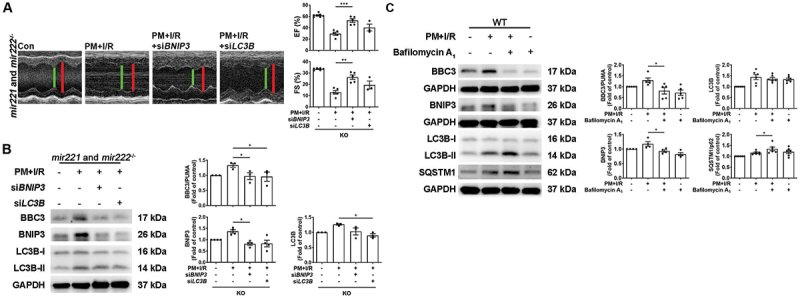
Knockdown of *Bnip3* and *Lc3b* expression improves cardiac function and reduces apoptosis-related proteins. (A and B) *mir221-* and *mir222-*ko mice were preconditioned with PM for 24 h. Then, 25 min after occlusion, si*BNIP3* or si*LC3B* were uniformly intramuscularly injected into the left ventricular marginal zone. EF and FS results were obtained by echocardiography to assess cardiac function (*n* = 4 mice per group). The expression levels of BBC3/PUMA, BNIP3, and MAP1LC3B/LC3B were also examined by western blot (*n* = 3 mice per group). (C) WT mice were intratracheally injected with PM and intraperitoneally injected with bafilomycin A_1_ (2.5 mg/kg) for 24 h, followed by 30 min of ischemia and 3 h of reperfusion. The levels of BBC3/PUMA, BNIP3, MAP1LC3B/LC3B, and SQSTM1/p62 were measured by western blot (*n* = 4-5 mice per group).

*Mir221-* and *Mir222-*overexpressing TG mice were used to further confirm the critical role of *Mir221* and *Mir222* in PM+I/R-induced cardiac injury. Moreover, myocardial *Mir221* and *Mir222* levels were significantly greater in *Mir221-* and *Mir222-*TG mice subjected to PM+I/R than in WT mice subjected to the same treatment (Figure 7J). Echocardiography showed that the EF and FS were greater in the PM+I/R-treated *Mir221-* and *Mir222-*TG mice than in the PM+I/R-treated normal mice, which was similar to the findings in the ADSC-Exos-treated group (Figure 7K). Compared with those in the PM+I/R-treated WT group, the LDH and TNNI levels in the PM+I/R-treated *Mir221-* and *Mir222-*TG group were significantly lower (Figure 7L). DHE and MitoSox Red staining indicated that *Mir221-* and *Mir222-*TG mice exhibited low ROS levels after PM+I/R treatment, and these results were similar to those obtained for ADSC-Exos-treated WT mice after PM+I/R treatment (Figure 7M, S2T, and S2U). In addition, western blot analysis indicated that *Mir221-* and *Mir222-*TG mice exhibited low expression of BNIP3 and MAP1LC3B/LC3B after PM+I/R treatment, which was similar to ADSC-Exos-treated WT mice after PM+H/R treatment (Figure 7N). Moreover, *Mir221-* and *Mir222-*TG mice also exhibited low expression of p-TRP53/p53 and BBC3/PUMA after PM+I/R treatment, as detected by western blot analysis, and reduced cell apoptosis, as detected by TUNEL assays. These results were consistent with those obtained for ADSC-Exos-treated WT mice after PM+I/R treatment (Figures 7O,P). These findings suggested that ADSC-Exos prevented myocardial PM+I/R injury through *Mir221* and *Mir222 in vivo*. To comprehensively elucidate the regulation of BNIP3, MAP1LC3B/LC3B, and BBC3/PUMA by *Mir221* and *Mir222*, we knockdown *Bnip3* and *Lc3b* in *mir221-* and *mir222-*KO mice. Transfection with si*BNIP3* significantly increased EF and FS in *mir221-* and *mir222-*KO mice subjected to PM+I/R treatment, indicating improved cardiac function (Figure 8A). Additionally, western blot showed si*BNIP3* and si*LC3B* significantly reduced BBC3/PUMA expression levels in PM+I/R-treated *mir221-* and *mir222-*KO mice (Figure 8B). Moreover, bafilomycin A_1_ treatment significantly reduced BBC3/PUMA expression in PM+I/R-treated WT mice (Figure 8C), showing that inhibition of autophagy contributes to reducing apoptosis in PM+I/R-induced cardiac injury. Taken together, these results indicated that I/R combined with PM exposure leads to increased ROS production, impaired mitochondrial function, maladaptive mitophagy, and increased apoptosis, ultimately leading to cardiac damage.

Statistical significance was defined as *p < 0.05, **p < 0.01, and ***p < 0.001.

## Discussion

Epidemiological studies have revealed a significant association between short-term exposure to environmental PM and increased daily mortality and hospitalization from cardiovascular diseases [28,29]. In the present study, exposure to PM under I/R conditions exacerbated mitochondrial oxidative stress, leading to mitochondrial fission, mitophagy, and apoptosis. In addition, ADSC-Exos exert their therapeutic effect by effectively alleviating the above effects, thereby mitigating mitochondrial dysfunction and cell apoptosis, ultimately restoring cardiac function. The present results provided evidence that *Mir221-* and *Mir222-*enriched ADSC-Exos reduce mitophagy and apoptosis through the BNIP3-MAP1LC3B/LC3B-BBC3/PUMA pathway and contribute to improving cardiac dysfunction.

Oxidative stress is essential for myocardial damage during acute I/R and chronic remodeling after myocardial infarction [30,31]. PM reduces cell viability and promotes apoptosis by increasing intracellular ROS production and activating the MAPK and NFKB/NF-κB signaling pathways in rat cardiac H9c2 cells [32,33]. The present study showed that PM or I/R treatment alone significantly increased mitochondrial ROS and total ROS production, and PM exacerbated I/R-induced mitochondrial ROS and total ROS. Consistent with the *in vivo* studies, PM exacerbated H/R-induced ROS production and apoptosis in cultured cardiomyocytes. Due to the limitation of the large number of cells required for this study, functionally similar H9c2 cells were used instead of primary cardiomyocytes, but our *in vitro* data are consistent with the *in vivo* findings. Importantly, oxidative stress indicates mitochondrial abnormalities [30]. Mitochondrial quality control includes mitochondrial biogenesis, mitochondrial dynamics (fission/fusion), and mitophagy [34]. In the rat aorta, PM increases SOD2 and mitochondrial fission proteins (DNM1L/Drp1 and FIS1) but reduces the expression of mitochondrial fusion proteins (MFN2 and OPA1) [16]. Previous studies have shown that mitophagy can clear damaged mitochondria after I/R injury, and mitophagy is often viewed as a defensive or adaptive process [35,36]. However, excessive or uncontrolled mitophagy (maladaptive) may leave cells without enough healthy or functioning mitochondria to produce ATP, thus limiting cell viability [37]. Increasing evidence suggests that I/R may cause an imbalance in the autophagy process, leading to additional cytotoxic damage that may induce cell death [10,38]. Moreover, PM activates mitophagy-related proteins, including LC3, SQSTM1/p62, PINK, and PRKN/parkin, in the rat aorta [16]. The present study demonstrated that PM+H/R decreased mitochondrial length and increased the expression of the DNM1L/Drp1 and MFF mitochondrial fission-related proteins. Moreover, the combination of PM and H/R increased the number of ALs and the expression of the BNIP3 and MAP1LC3B/LC3B mitophagy-related proteins. The degree of apoptosis was significantly reduced when mitochondrial ROS production, mitochondrial division, and mitophagy production were inhibited by MitoTEMPO, Mdivi-1, and bafilomycin A_1_, respectively. These results suggested that mitochondrial oxidative stress is enhanced after PM+H/R treatment, leading to mitochondrial damage, increased mitochondrial fission, and mitophagy. Thus, the cascade of events driven by oxidative stress ultimately promotes apoptosis.

*Mir221* and *Mir222* have been reported to be closely related to inflammation and the pathogenesis of cardiovascular diseases [22,39]. Both miRNAs counteract myocardial fibrosis in pressure overload-induced heart failure [22]. The levels of *Mir221* and *Mir222* in circulating progenitor cells may provide expected pharmacological effects [40]. We have previously reported that the expression of *Mir221* and *Mir222* in cardiac tissue is significantly reduced after I/R injury [20]. In the present study, we found that compared to I/R (H/R) or PM alone, combined treatment with I/R (H/R) and PM significantly reduced the expression of *Mir221* and *Mir222*. We have previously demonstrated that ADSC-derived conditioned media or exosomes containing high levels of *Mir221* and *Mir222* decrease I/R-induced cardiac injury [20,27]. Exosomes derived from various cells are considered a novel drug delivery method. For instance, mesenchymal stem cell (MSC)-derived exosomes containing miRNAs and proteins are considered alternatives to cell therapy and play a promising role in cardiac regeneration and repair [41]. The transfer of *Mir221* and *Mir222* via vascular smooth muscle cells – derived exosomes from diabetes affected vascular inflammation [42]. Few articles have reported the relationships among *Mir221* and *Mir222*, mitophagy, and apoptosis. Elevated levels of *Mir221* and *Mir222* in glioblastoma promote cell survival, while reducing the levels of these miRNAs leads to increased apoptosis through the upregulation of BBC3/PUMA [43]. Another study has shown that *Mir221* and *Mir222* reduces the expression of the MAP1LC3B/LC3B autophagy protein and inhibits apoptosis in PTC cells [23]. *Mir221* significantly decreases cardiac H/R-induced autophagosome formation and reduces the LC3-II:I ratio [44,45]. Extracellular vesicle-miRNA (hsa- *Mir221*-3p) levels are downregulated after PM exposure, and patients exhibit inflammation during pregnancy [46]. The present study demonstrated that an increase in *Mir221* and *Mir222* significantly reduced the expression of BNIP3, MAP1LC3B/LC3B, and BBC3/PUMA, indicating the regulatory role of *Mir221* and *Mir222* in mitophagy and apoptosis in PM+I/R (H/R)-induced cardiac injury.

This study clarified the regulatory role of *Mir221* and *Mir222* through the BNIP3-MAP1LC3B/LC3B-BBC3/PUMA pathway, but the exact molecular mechanism remains to be further studied. However, it is necessary to verify whether *Mir221* and *Mir222* regulates apoptosis through the PRKAA1/AMPK-CDKN1B/p27 pathway. The present study showed that the PRKAA1/AMPK-CDKN1B/p27 pathway was induced under PM+I/R (or H/R) conditions, whereas treatment with ADSC-Exos or *Mir221* and *Mir222* mimics reversed these changes. Furthermore, PM+I/R (H/R)-induced apoptosis was reversed by treatment with PRKAA1/AMPK and CDKN1B/p27 inhibitors. Previous studies have shown that *Mir221* and *Mir222* inhibits CDKN1B/p27 expression by targeting the 3’UTR [47]. Apo E inhibits smooth muscle cell proliferation by regulating CDKN1B/p27 expression via *Mir221* and *Mir222* [48]. Notably, the present study revealed that in WT and *mir221-* and *mir222-*KO mice subjected to PM+I/R, the administration of ADSC-Exos or *Mir221-* and *Mir222-*mimics significantly reduced p-PRKAA1/AMPK and p-CDKN1B/p27 expression (Figure S4E). Moreover, *Mir221-* and *Mir222-*TG mice also expressed low levels of p-PRKAA1/AMPK and p-CDKN1B/p27, similar to what was observed in PM+I/R+ADSC-Exos-treated WT mice (Figure S4F). These findings suggested that ADSC-Exos may reduce PM+I/R-induced apoptosis by targeting the *Mir221* and *Mir222*-PRKAA1/AMPK-CDKN1B/p27 signaling cascade.

The mechanism of how *Mir221* and *Mir222* regulates ROS production and mitochondrial fission remains to be determined. Previous studies have shown that *Mir221* and *Mir222* directly inhibits the expression of endothelial nitric oxide synthase (eNOS) or SOD2, reducing the production of nitric oxide (NO) and ROS, respectively [39,49]. Additional studies are required to determine whether *Mir221* and *Mir222* regulates ROS production by targeting eNOS and SOD2. Our results demonstrate significant differences in the effects of ADSC-Exos on cardiac function depending on the presence or absence of *Mir221* and *Mir222* within ADSC-Exos (Figure S5).

Overall, the present findings demonstrated that PM aggravates I/R (H/R)-induced cardiac injury. *Mir221-* and *Mir222-*enriched ADSC-Exos reduce PM+I/R-induced mitophagy and apoptosis by downregulating BNIP3, MAP1LC3B/LC3B, and BBC3/PUMA. The present study provides a new treatment for PM+I/R-induced cardiac injury.

## Materials and methods

### PM preparation for mouse myocardial ischemia-reperfusion (I/R)

SRM 2786 fine particulate matter (<4 microns) is a standard reference material (SRM) that is used according to the US Environmental Protection Agency/EPA national standard for ambient air quality [50]. SRM 2786 was suspended in PBS (Omics Bio, IB3012) at 10 mg/mL concentration and sonicated for 10 min to reduce particle aggregation. All experiments using C57BL/6J mice, *mir221-* and *mir222-*KO mice, and *Mir221*- and *Mir222*-overexpressing transgenic mice were performed in accordance with the Guidelines for the Use of Laboratory Animals of the National Institutes of Health and approved by the Animal Ethics Committee of National Taiwan University (IACUC No. 20180426). Mice were anesthetized by inhalation of 4% isoflurane mixed with 100% oxygen and injected intratracheally with a solution of 200 μg PM in 50 μL PBS. After 24 h, the mice were anesthetized again, and the left coronary artery was ligated with 8–0 nylon suture for 30 min. The occlusion was confirmed by observing the pale myocardium of the left ventricle. The ligation was then removed, and the animals were sacrificed after 3 h of blood flow. All efforts were made to minimize suffering; mice were euthanized through isoflurane overdose, followed by cardiac excision.

### Echocardiography

Echocardiography was performed using a small animal high-resolution ultrasound system (Prospect, S-Sharp, Taiwan) equipped with a 40 MHz single-element transducer. M-mode images obtained from the parasternal long axis view (PLAX) were used to assess fractional shortening (FS) and ejection fraction (EF) as parameters for estimating systolic function. The detailed procedures are described in the Supplemental Material.

### Cultured cardiomyocytes for hypoxia-reoxygenation (H/R)

Embryonic rat cardiomyocyte-derived H9c2 cardiac myoblasts were obtained from the American Type Culture Collection/ATCC (CRL-1446). The cells were cultured in Dulbecco’s modified Eagle’s medium (DMEM; Gibco 12,800–017) and exposed to PM at concentrations of 10 µg/mL or 50 µg/mL for 6 h. For hypoxia treatment, the cells were incubated under hypoxic conditions (1% O_2_) at 37°C for 6 h, followed by incubation under normoxic reoxygenation conditions for 12 h.

### Exosome isolation

Human adipose-derived stem cells (ADSCs, 5 × 10^5^ cells) were seeded into a 10-cm dish. After 24 h of incubation, the conditioned culture medium was collected and centrifuged to remove cells and cell debris. The supernatant (10 mL) was transferred to a sterile vessel, and 2 mL of ExoQuick-TC Exosome Precipitation Solution (System Biosciences, EXOTC50A–1) was added. The mixture was refrigerated overnight and then centrifuged at 10,000 × g for 30 min at 4°C. The resulting pellets were washed with PBS and subsequently suspended in PBS. The morphological characteristics of ADSC-Exos were examined through transmission electron microscopy (TEM). The protein concentration was determined using a BCA protein assay kit (Millipore 71,285-3CN). The isolated ADSC-Exos were stored at −80°C for subsequent use. Examining the characteristics of ADSC-Exos was provided in the supplementary file (Figure S3B to S3D).

### MTT assay, TUNEL assay, ANXA5/annexin V-FITC staining, and propidium iodide (PI) staining

The degree of cell viability was determined by a MTT (3-[4,5-dimethylthiazol-2-yl]-2,5-diphenyltetrazolium bromide) assay. The cells were incubated with MTT solution (BIONOVAS, AM0815–0001) at a final concentration of 0.5 mg/mL at 37°C for 1.5 h. Apoptotic cells were detected using terminal deoxynucleotidyl transferase dUTP nick end labeling (TUNEL) staining (*In Situ* Cell Death Detection Kit; Roche Molecular Biochemicals 11,684,817,910) and an ANXA5-FITC-PI staining detection kit (BD Biosciences 640,914). The cells were incubated with TUNEL reagent for 1 h at 37°C in the dark. Slides were counterstained with DAPI to visualize all nuclei and observed under a fluorescence microscope. H9c2 cells were suspended in 100 μL of binding buffer containing 2.5 μL of FITC and 5 μL of PI (100 μg/mL), followed by incubation in the dark at 4°C for 15 min. Cell death was then assessed by flow cytometry.

### MitoSOX red, DCFH-DA, and DHE staining

Mitochondrial ROS levels were detected using the MitoSOX Red (Invitrogen, M36008) mitochondrial superoxide indicator. The cells were treated with 1 μM MitoSox Red for 15 min at 37°C. TO-PRO-3 (100 nM; Thermo Fisher Scientific, T3605), a dead cell indicator, was added prior to MitoSox Red analysis using an LSRFortessa flow cytometer (BD Biosciences). Intracellular levels of oxidative radicals and superoxide anions were determined using 2“,7”-dichlorodihydrofluorescein diacetate (DCFH-DA; Thermo Fisher Scientific, C369) and dihydroethidium (DHE) (Invitrogen, D11347). Cardiomyocytes were cultured on coverslips, treated with PM+H/R, and then incubated with DCFH-DA (10 μM) for 30 min at 37°C in the dark. The conversion of DCFH-DA to a fluorescent DCF product was observed using fluorescence microscopy. Five microliters of PI solution (100 μg/mL) was added before flow cytometric analysis of DCFH-DA. PI enters damaged or ruptured cell membranes when the cell dies, providing a method to distinguish between live and dead cells. Cardiomyocytes were incubated in medium containing DHE (5 μM) at 37°C for 15 min. The oxidation of DHE by intracellular superoxide anions produces ethidium, which fluoresces red at 535 nm and is subsequently imaged by fluorescence microscopy. DiOC_6_(3) (90 nM; Thermo Fisher Scientific, D273), a lipophilic dye selective for mitochondria in living cells, was added prior to DHE analysis by flow cytometry.

### Acridine orange (AO) staining

The cells were briefly washed with PBS and incubated with 1 μg/mL AO hydrochloride solution (Invitrogen, A1301) for 5 min at RT. AO is a fluorophore that accumulates in protonated form within acidic vesicular organelles, including ALs. AO dimerizes at high concentrations, resulting in a metachromatic shift from green to red.

### Mitophagy assay using mito-keima

Transfection of mt-Keima red (MBL, AM-V0259) was performed according to the manufacturer’s instructions. H9c2 cells were transfected with mt-Keima plasmid (1 μg/mL) in serum-free medium at 37°C for 24 h, then incubated in a hypoxic environment (1% O_2_ and 5% CO_2_) for 6 h, followed by oxygenation for 12 h. The cells were observed under a fluorescent microscope. Mt-Keima is a pH-sensitive fluorescent protein. When mitochondria are delivered to acidic lysosomes, their excitation spectrum changes from 405 nm to 561 nm and emits in the wavelength range of 570 to 706 nm, showing a color change from green to red.

### JC-1 analysis

5,5“,6,6”-Tetrachloro-1,1“,3,3”-tetraethylbenzimidazolyl-iodocarbocyanine (JC-1, BD biosciences 551,302) was used to analyze changes in the mitochondrial membrane potential. When the mitochondrial membrane potential is low, JC-1 emits green fluorescence as a monomer, and when the mitochondrial membrane potential is high, it aggregates and emits red fluorescence. After various treatments, the cells were incubated with JC-1 (2 μM) for 30 min at 37°C in the dark. Cell fluorescence was monitored using an SP2 microscope (Leica, Wetzlar, Germany) and flow cytometry.

### Adenosine triphosphate (ATP) production and seahorse test

Cellular ATP levels were assessed using an ATP assay kit (Invitrogen, A-22066). The cells were lysed and centrifuged (12,000 × g) for 5 min at 4°C. Subsequently, the supernatants were collected and analyzed for ATP levels.

Mitochondrial respiration was assessed with the Seahorse XF Cell Mito Stress Test Kit (Agilent 103,010–100), which directly measures the oxygen consumption rate (OCR) using a Seahorse XFe24 Extracellular Flux Analyzer (Agilent). In brief, cells (1 × 10^4^ cells/well) were seeded into 24-well plates (Agilent). After washing the cells twice with PBS, the medium was changed to XF base medium (pH 7.4 DMEM plus 1 mM pyruvate [Biological Industries, 03–042-1B], 2 mM glutamine [Biological Industries, 03–020-1B], and 10 mM glucose), followed by incubation at 37°C in a non-CO_2_ incubator for 1 h. The cells were then injected sequentially through ports in the XF Cell Mito Stress Test Kit (Agilent 103,010–100) with oligomycin (1 μM), FCCP (2 μM), and rotenone (0.5 μM) combined with antimycin (0.5 μM) to assess the ATP-related OCR, maximal OCR and nonmitochondrial OCR.

### MitoTracker staining

Mitochondrial length was examined using MitoTracker (Invitrogen, M22425) staining. After the various treatments were completed, the cardiomyocytes were incubated with 400 nM MitoTracker for 1 h at 37°C in the dark. Visualization was performed using total internal reflection fluorescence microscopy (TIRF, Zeiss) and DIC optics, and mitochondrial length was measured using ImageJ software.

### Transmission electron microscopy (TEM)

The mitochondrial ultrastructure and mitophagy of cardiac tissue and H9c2 cells were observed by TEM. The samples were fixed in 2% glutaraldehyde and 2% paraformaldehyde in 0.1 M PBS overnight at 4°C. The fixed specimens were dehydrated in ethanol and then embedded in epoxy resin. Ultrathin sections were double-stained with uranyl acetate and lead citrate, followed by examination with a Hitachi H700 electron microscope (Hitachi, Tokyo, JP).

### Western blot analysis

The heart was pulverized using liquid nitrogen and subsequently homogenized in RIPA buffer (TOOLS, 1002-W), a commercially available product containing 154 mM NaCl, 1% Nonidet *p*-40 (TOOLS, 9016-45-9), 0.8 mM EDTA, 65.2 mM Tris base, pH 7.8 and 0.25% sodium deoxycholate (TOOLS, 302-95-4) along with a protease inhibitor cocktail (Thermo Fisher Scientific 78,442). The cell lysate was prepared in RIPA buffer containing 1% protease inhibitor cocktail at 4°C for 1 h. The supernatant was retained after centrifuging the lysate at 14,000 × g for 20 min at 4°C. Samples with equal amounts of protein (30 μg) were electrophoresed on SDS‒polyacrylamide gels and transferred to PVDF membranes (Millipore, IPVH00010). The membranes were probed overnight at 4°C with the following primary antibodies: DNM1L/Drp1, MFF, BECN1/Beclin 1, BNIP3, MAP1LC3B/LC3B, p-TRP53/p53, CASP3, CASP9, p-PRKAA1/AMPK (1:2000 dilution; Cell Signaling Technology, 8570; 84580; 3495; 3769; 2775; 9284; 9664; 9508; 2535), BCL2 (1:1000 dilution; BD Biosciences 610,539), BBC3/PUMA (1:2000 dilution; Abcam, AB9643), and ACTB/β-actin (1:10000 dilution; Proteintech 66,009–1). The membranes were then incubated with horseradish peroxidase-conjugated goat anti-mouse or anti-rabbit IgG secondary antibodies (Jackson, 111-035-003; 115-035-003). GAPDH (Proteintech 60,004–1) was used as the internal control. The intensity of the bands was quantified using ImageJ software (NIH, MD, USA).

### Mitochondrial isolation assay

The Thermo Mitochondrial Isolation Kit (Thermo Fisher Scientific 89,874) was used to isolate mitochondrial and cytoplasmic fractions from H9c2 cell lysates according to the manufacturer’s instructions. The mitochondrial and cytoplasmic fractions were stored at −80°C, after which the cellular location and protein expression levels were evaluated by western blot analysis.

### Double immunofluorescence staining

Cells on coverslips were washed with PBS, fixed with 4% paraformaldehyde for 15 min at room temperature, and permeabilized for 1 min with 0.1% Triton X-100. The cells were blocked with 1% bovine serum albumin (BSA; Sigma-Aldrich 126,593) in PBS for 1 h at room temperature, followed by were incubation with anti-BNIP3 (Cell Signaling Technology, 3769) or anti-LC3B (Cell Signaling Technology, 2775) primary antibodies (1:200 dilution in PBS containing 1% BSA) overnight at 4°C. After washing with PBS, the cells were incubated with anti-rabbit Alexa Fluor® 488 (Invitrogen, A27034; 1:200 dilution in PBS containing 1% BSA) for 1 h at room temperature. The cells were exposed to COX4/COX IV (Thermo Fisher Scientific, A21348) diluted 1:200 in PBS containing 1% BSA overnight at 4°C. After washing 3 times with PBS, the cells were incubated with anti-mouse Alexa Fluor® 647 (Invitrogen, A20173) for 1 h at room temperature in the dark. The cells were then counterstained with DAPI (1 μg/mL) for 5 min. An SP8 inverted confocal microscope (Leica, Wetzlar, Germany) was used for imaging and analysis.

### RNA isolation and quantitative real-time PCR

According to the manufacturer’s protocol, total RNA was isolated from cells or frozen mouse ventricular tissues using TRIzol reagent (Thermo Fisher Scientific 78,442). RNA quantification was performed using a NanoDrop™ 2000 spectrophotometer (Thermo Fisher Scientific). The expression levels of *Mir221* and *Mir222* were quantified using commercially available TaqMan MicroRNA assay kits for *Mir221* (000524), *Mir222* (002276), and *RNU6-6P/RNU6B* (001973). For the real-time PCR, TaqMan™ Universal PCR master mix without UNG was used, and the reactions were analyzed by a QuantStudio™ 3 Real-Time PCR System (Applied Biosystems).

### Transient transfection

H9c2 cells were seeded into 6-well plates at a density of 2 × 10^5^ cells per well and were grown to 70–80% confluence. The cells were transfected with specific *Mir221-* and *Mir222-*mimics or inhibitors (100 nM/well; Dharmacon) to overexpress or knockdown *Mir221* and *Mir222*, respectively, using Lipofectamine 3000 reagent (Invitrogen, L3000–015) according to the manufacturer’s protocol. The cells were then subjected to MAP1LC3B/LC3B and BNIP3 expression analysis.

### Dual-luciferase reporter assay

The wild-type (WT) and mutant (MUT) *Bnip3* (or *Lc3b*) 3”-UTR luciferase reporter gene plasmids were produced (Promega 0,000,389,032). Cells were transfected with Mir-NC or *Mir221-* and *Mir222-*mimics along with the WT or MUT *Bnip3* (or *Lc3b*) 3”−UTR reporter plasmids using Lipofectamine 3000 reagent for 48 h.

### Statistical analysis

The data were analyzed using SPSS 19.0 statistical software (SPSS, Chicago, IL, USA). The measurement data are presented as the mean ± standard error of the mean (SEM). One-way analysis of variance (ANOVA) was conducted among multiple groups. *p* < 0.05 indicated statistical significance.

## Supplementary Material

Autophagy Supplementary Material R5...docx

## Data Availability

All data are included in the manuscript and supplementary information. Additional materials related to this paper are available upon reasonable request from the corresponding authors.
