# Supplementary material for: Mir221- and Mir222-enriched adsc-exosomes mitigate PM exposure-exacerbated cardiac ischemia-reperfusion injury through the modulation of the BNIP3-MAP1LC3B-BBC3/PUMA pathway
Source: Autophagy. 2024 Sep 8;21(2):374–93. doi: 10.1080/15548627.2024.2395799 (PMC11760231; doi:10.1080/15548627.2024.2395799)
Supplement: Autophagy Supplementary Material R5...docx [file KAUP_A_2395799_SM4161.docx]

**Supplementary figures**

**
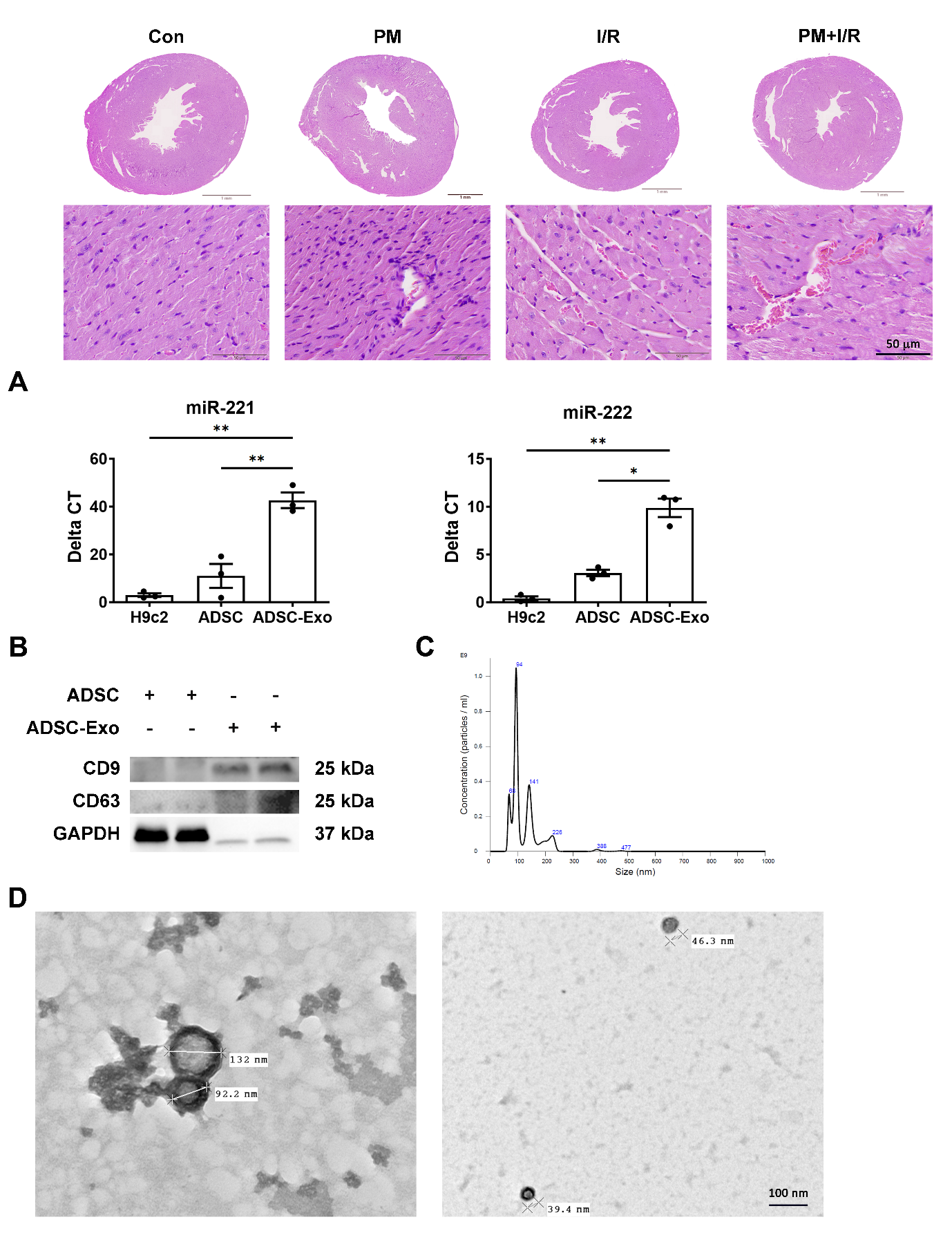
**

**Figure S1.** Cardiac morphology was observed by hematoxylin and eosin (HE) staining. WT mice were pretreated with PM (10 mg/kg) for 24 h, followed by 30 min of myocardial ischemia, and then by 3 h of reperfusion during I/R. The morphology of the cross-section of the heart was observed with HE staining. No significant damage to cardiac morphology was observed in PM+I/R-treated hearts. Scale bar in upper figures: 1 mm; scale bar in bottom figures: 50 μm.


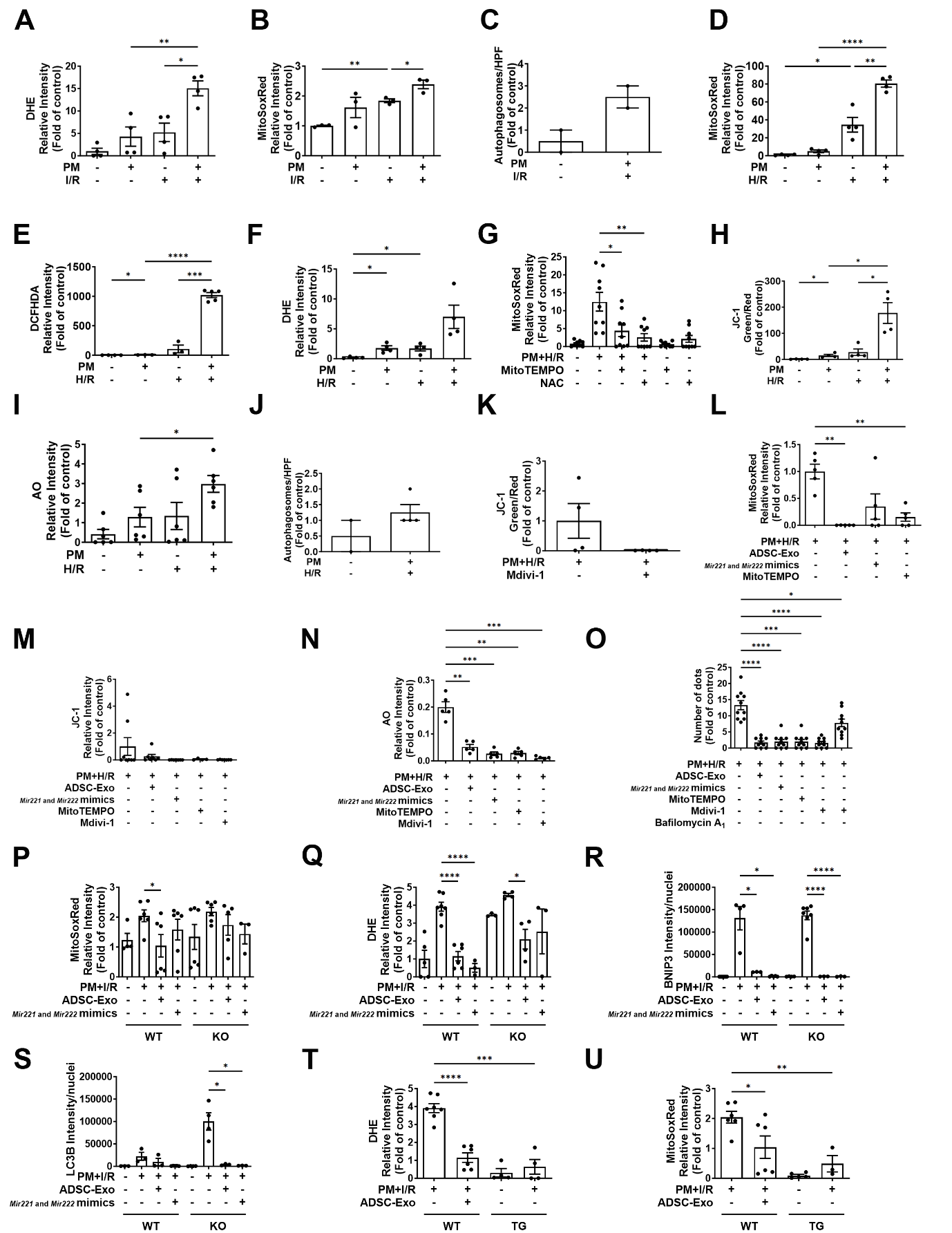


**Figure S2.** Quantitative analyses. The data presented in the figure are related to: (**A**) Figure 1F, (**B**) Figure 1G, (**C**) Figure 1H, (**D**) Figure 2E, (**E**) Figure 2G, (**F**) Figure 2I, (**G**) Figure 2K, (**H**) Figure 3C, (**I**) Figure 3I, (**J**) Figure 3K, (**K**) Figure 3L, (**L**) Figure 6A, (**M**) Figure 6C, (**N**) Figure 6H, (**O**) Figure 6I, (**P**) Figure 7D, (**Q**) Figure 7E, (**R and S**) Figure 7G, (**T and U**) Figure 7M of the main manuscript.


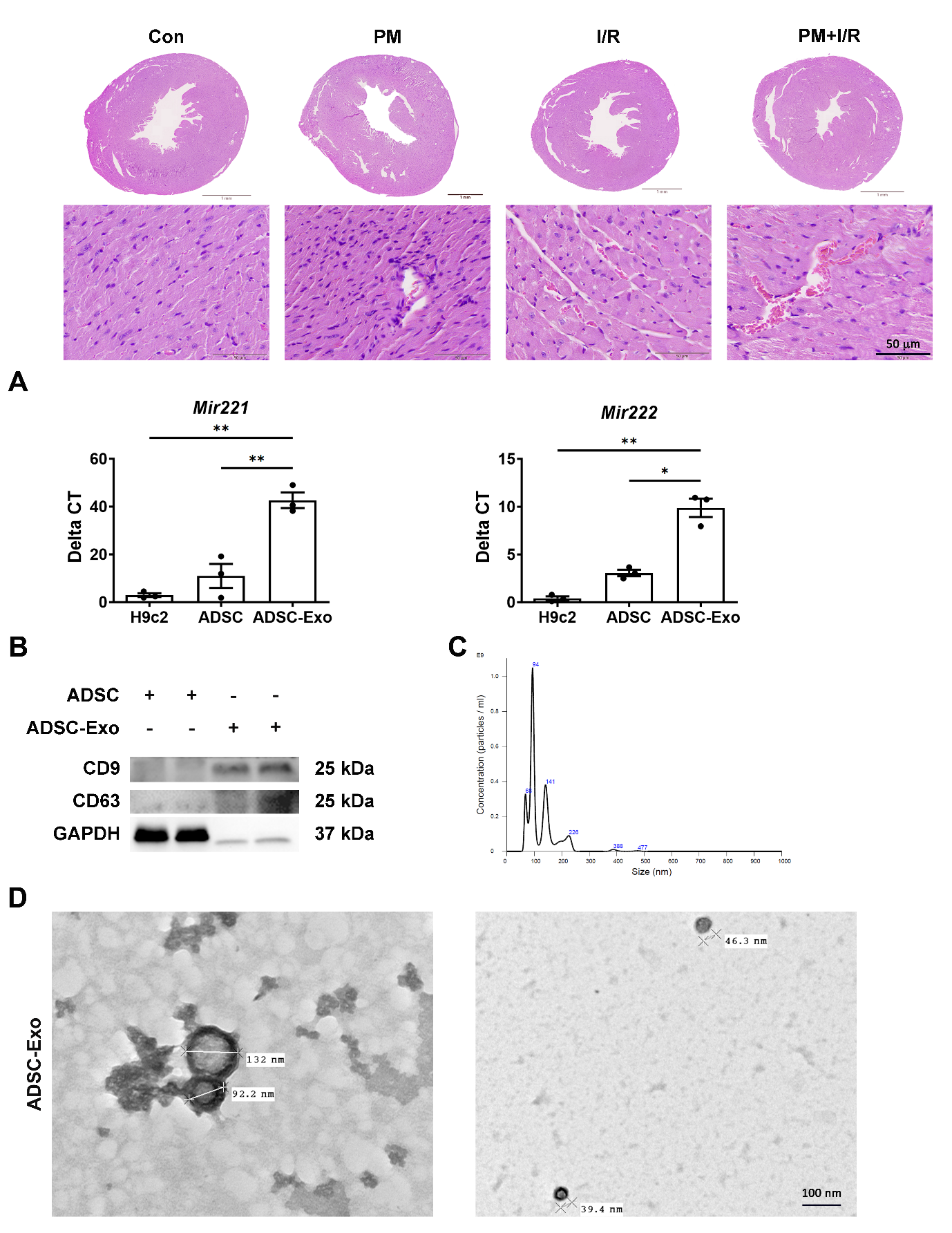


**Figure S3.** *Mir221-* and *Mir222-*expression and characterization of ADSC-Exos. (**A**) The content of *Mir221* and *Mir222* in ADSC cells was markedly higher than that in H9c2 cells. Furthermore, the content of *Mir221* and *Mir222* was significantly higher in ADSC-Exos than in ADSC cells (n=3-4). Data are expressed as mean ± SEM; One Way ANOVA. Statistical significance was defined as *P < 0.05, **P < 0.01. (**B**) Western blot analysis was used to evaluate the expression of exosome biomarkers CD9 and CD63 in ADSC cell lysate and ADSC-Exos, respectively. The levels of CD9 and CD63 were strongly expressed in ADSC-Exos. (**C**) ADSC-Exos were subjected to NTA analysis. The average size of ADSC-Exos was 122 nm, and the mode value (major peak) was 94 nm. (**D**) The morphology and size of ADSC-Exos were observed by transmission electron microscopy (TEM). Scale bar: 100 nm.


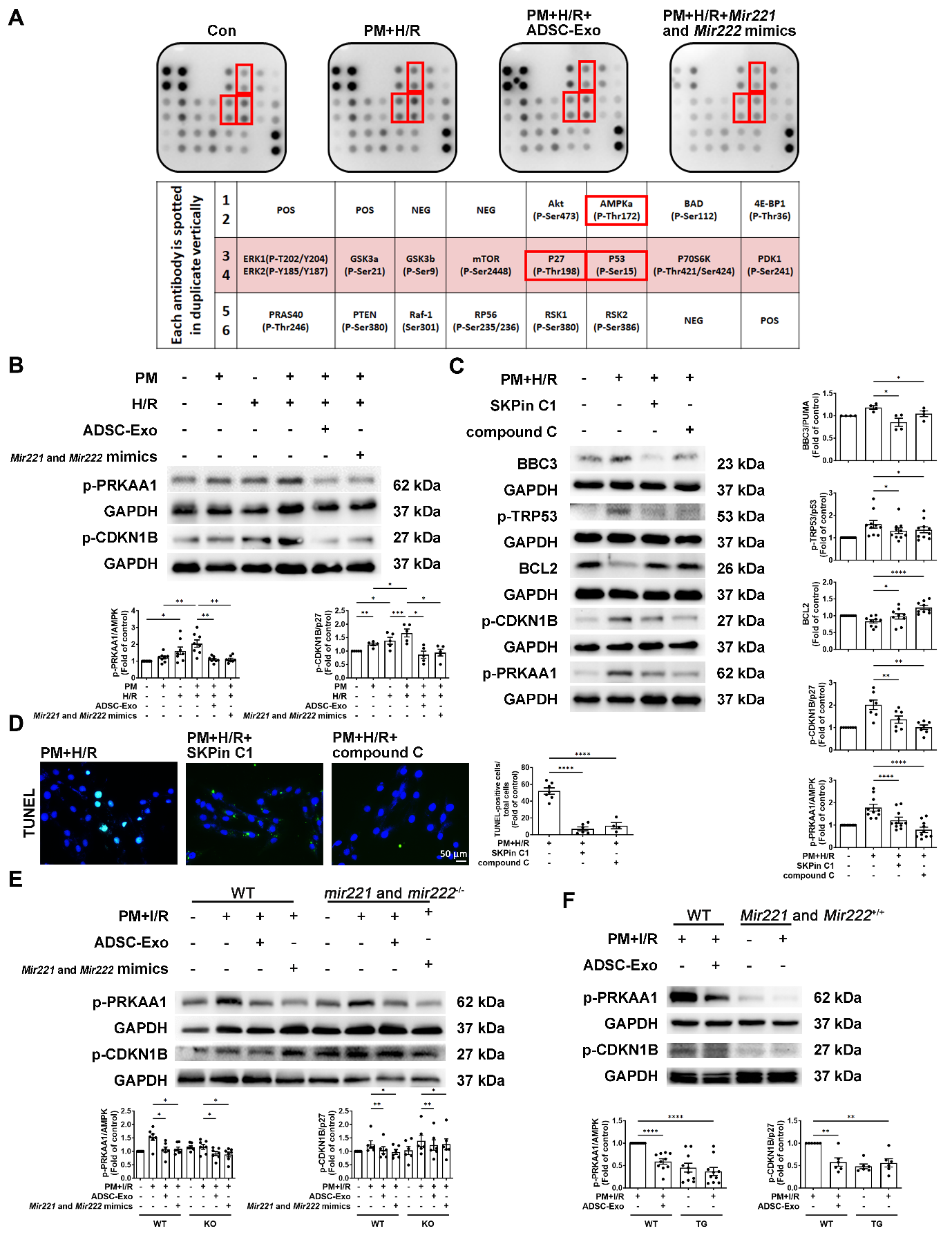


**Figure S4.** Explore the involvement of the AKT pathway in PM+I/R enhancing apoptosis and ADSC-Exos attenuating this effect. To investigate whether other signaling molecules are involved in PM+I/R-enhanced apoptosis, the AKT pathway phosphorylation array was used to identify specific pathways. H9c2 cells were pretreated with PM (50 μg/mL) for 6 h and treated with H/R conditions (6 h of hypoxia followed by 12 h of reoxygenation). (**A**) A phosphorylation proteomic array was used to verify the effect of ADSC-Exos on signaling mechanisms related to PM+H/R-induced cardiac injury. Differential expression array data showed that PM+H/R treatment induced upregulation of apoptosis regulatory factors, such as PRKAA1/AMPK, CDKN1B/p27, and TRP53/p53. (**B**) H9c2 cells were exposed to ADSC-Exos (2 µg/mL) or *Mir221-* and *Mir222-*mimics (100 nM) for 24 h, followed by PM (50 µg/mL) treatment and H/R. The effects of ADSC-Exos and *Mir221-* and *Mir222-*mimics transfection on the expression of p-PRKAA1/AMPK and p-CDKN1B/p27 were examined. Significant changes in p-PRKAA1/AMPK and CDKN1B/p27 between PM+H/R treatment and ADSC-Exos were detected by western blot (n=5-8). (**C and D**) To elucidate whether the effect of ADSC-Exos on apoptosis in PM+H/R-treated H9c2 cells was regulated by the PRKAA1/AMPK-CDKN1B/p27 pathway, H9c2 cells were treated with a CDKN1B/p27 inhibitor (SKPin C1) or with an PRKAA1/AMPK inhibitor (compound C). H9c2 cells were pretreated with 10 μM SKPin C1 or with 10 μM compound C for 1 h before exposure to PM and H/R (6 h/12 h). (**C**) Cells treated with SKPin C1 or compound C reduced PM+H/R-induced p-TRP53/p53 and BBC3/PUMA expression while increasing BCL2 expression (n=4-10). (**D**) TUNEL assay detected the effects of SKPin C1 and compound C on PM+H/R-induced apoptosis. SKPin C1 and compound C treatment significantly reduced PM+H/R-induced apoptosis (n=4-7). Scale bar: 50 μm. (**E**) WT or *mir221-* and *mir222-*KO mice were pretreated with PM for 24 h, followed by 30 min of myocardial ischemia. Subsequently, 25 min after occlusion, ADSC-Exos (100 μg protein in 50 μL) or *Mir221-* and *Mir222-*mimics (100 nM) were uniformly injected into the border zone of the anterior wall and then perfused for another 3 h. The effects of ADSC-Exos and *Mir221-* and *Mir222-*mimics transfection on p-PRKAA1/AMPK and p-CDKN1B/p27 expression were examined by western blot in WT and KO mice. WT and KO mice treated with ADSC-Exos or *Mir221-* and *Mir222-*mimics significantly reduced p-PRKAA1/AMPK and p-CDKN1B/p27 expression compared with PM+I/R-treated mice (n=6-7). (**F**) WT and *Mir221-* and *Mir222-*TG mice were pretreated with PM for 24 h and then underwent I/R (30 min of ischemia followed by 3 h of reperfusion). Western blot was performed to assess the expression and quantification of p-PRKAA1/AMPK and p-CDKN1B/p27 in WT and TG mice. Compared with the PM+I/R-treated WT group, the levels of p-PRKAA1/AMPK and p-CDKN1B/p27 expression were significantly reduced in PM+I/R-treated TG mice (n=6-10). Data are expressed as mean ± SEM; one way ANOVA. Statistical significance was defined as *P < 0.05, **P < 0.01, and ***P < 0.001.


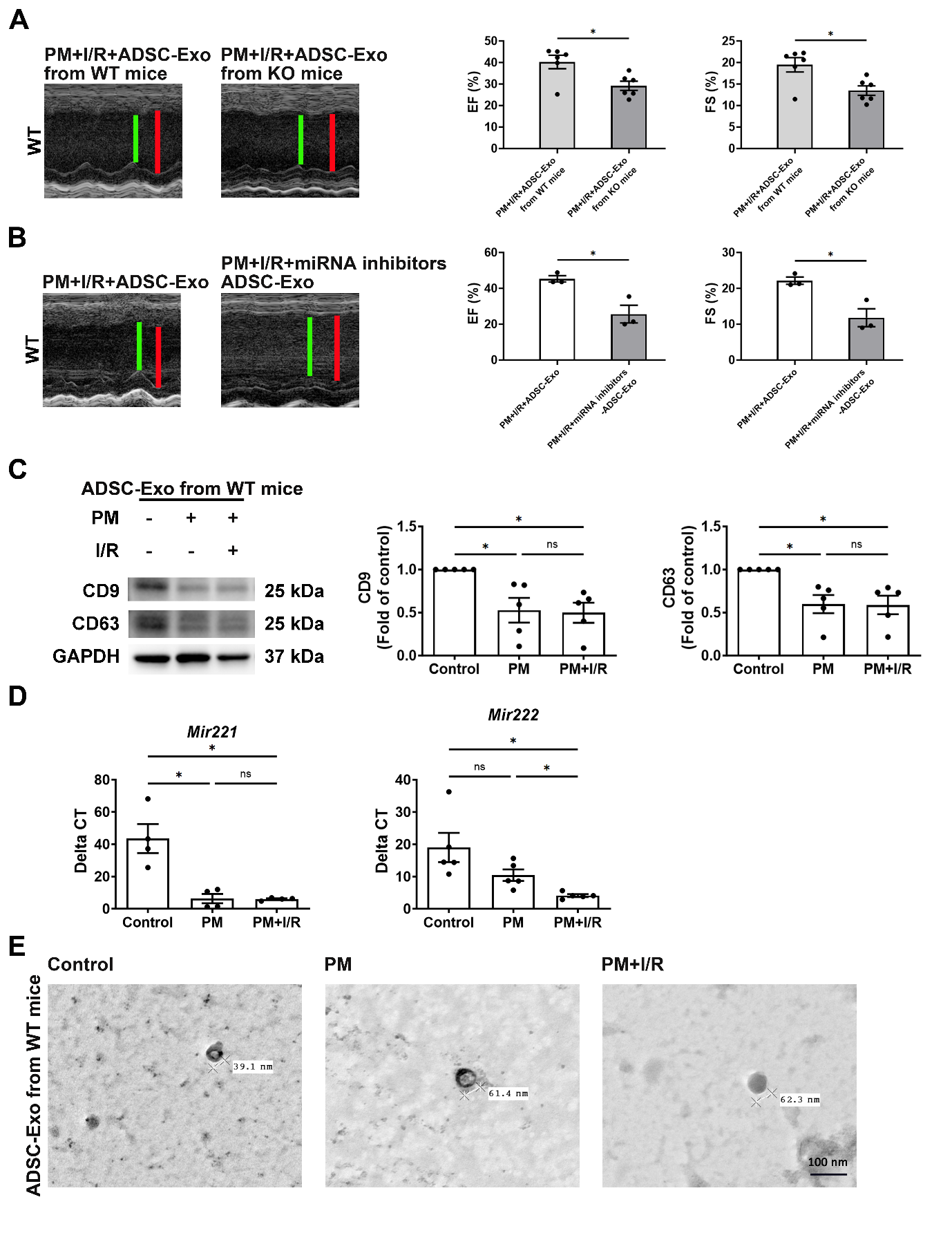


**Figure S5.** Comparison of ADSC-Exos from WT and *mir221-* and *mir222-*KO mice in PM+I/R-induced cardiac dysfunction. (**A**) Adipose-derived stem cells (ADSCs, 5×10^5^ cells) from WT or *mir221-* and *mir222-*KO mice were seeded into 10-cm culture dishes. After 24 h, exosomes were isolated by ExoQuick-TC Exosome Precipitation Solution. WT mice were pretreated with PM for 24 h, followed by 30 min of myocardial ischemia. Subsequently, ADSC-Exos isolated from WT or KO mice were uniformly injected into the border zone of the anterior wall, 25 min after occlusion. The mice were then perfused for an additional 3 h before measuring cardiac function using echocardiography. Results showed that ADSC-Exos from KO mice failed to improve PM+I/R-induced cardiac dysfunction (n=6 mice per group). Statistical significance was defined as *P < 0.05. (**B**) Human ADSCs were transfected with or without *Mir221-* and *Mir222-*inhibitors, and exosomes were isolated. WT mice were pretreated with PM for 24 h. Subsequently, *Mir221-* and *Mir222-*inhibitor-ADSC-Exos or ADSC-Exos was injected into the anterior wall of the left ventricle 25 min after occlusion. The ligation was then removed, and blood flow was restored for 3 h before assessing cardiac function using echocardiography. Results indicated that while ADSC-Exos reduced PM+I/R-induced cardiac dysfunction, *Mir221-* and *Mir222-*inhibitor-ADSC-Exos failed to improve PM+I/R-induced cardiac dysfunction (n=3 animals per group). ADSC-Exos collected after inhibiting *Mir221* and *Mir222* did not exhibit the same efficacy in reducing PM+I/R-induced cardiac dysfunction as observed with the original ADSC-Exos. Statistical significance was defined as *P < 0.05. (**C-E**) Study of ADSC-Exos characteristics in WT mice under PM and PM+I/R conditions. WT mice were divided into three groups: control, PM, and PM+I/R. ADSCs (5 × 10⁵ cells) obtained from these three groups of adipose tissue were cultured for 24 h, and exosomes were isolated using ExoQuick-TC Exosome Precipitation Solution. (**C**) Western blot analysis was conducted to evaluate the expression of CD9 and CD63 (exosome markers) (n=5). Levels of CD9 and CD63 were significantly reduced in WT mice treated with PM or PM+I/R. (**D**) qPCR was performed to measure the levels of *Mir221-* and *Mir222-*expression in RNA extracted from the three groups (n=5). The levels of *Mir221-* and *Mir222-*expression were markedly reduced in WT mice treated with PM or PM+I/R. (**E**) The morphology and size of ADSC-Exos were examined using transmission electron microscopy. Scale bar: 100 nm. Statistical significance was defined as *P < 0.05.

**Table S1**. List of echocardiographic variables analyzed for each group (mean ± SD).

IVS; d and IVS; s: interventricular septum in diastole and systole, LVID; d: diastolic left ventricular interior dimension; LVID; s: systolic left ventricular interior dimension, FS: fractional shortening, EDV: end-diastolic volume, ESV: end-systolic volume, EF: ejection fraction, CO: cardiac output.
